# Vegetables with Enhanced Iron Bioavailability—German Consumers’ Perceptions of a New Approach to Improve Dietary Iron Supply

**DOI:** 10.3390/nu15102291

**Published:** 2023-05-12

**Authors:** Ann-Kristin Welk, Clara Mehlhose, Diemo Daum, Ulrich Enneking

**Affiliations:** 1 Faculty of Agricultural Sciences and Landscape Architecture, Osnabrück University of Applied Sciences, Am Krümpel 31, 49090 Osnabrück, Germany; d.daum@hs-osnabrueck.de (D.D.); u.enneking@hs-osnabrueck.de (U.E.); 2 Marketing of Agricultural and Food Products, Department of Agricultural Economics and Rural Development, University of Göttingen, Platz der Göttinger Sieben 5, 37073 Göttingen, Germany; clara.mehlhose@uni-goettingen.de

**Keywords:** dietary supplements, functional food, iron biofortification, target group analysis, vitamin C, willingness to purchase

## Abstract

Iron deficiency is still widespread as a major health problem even in countries with adequate food supply. It mainly affects women but also vegans, vegetarians, and athletes and can lead to various clinical pictures. Biofortification of vitamin C-rich vegetables with iron may be one new approach to face this nutritional challenge. However, so far, little is known about the consumer acceptance of iron-biofortified vegetables, particularly in developed countries. To address this issue, a quantitative survey of 1000 consumers in Germany was conducted. The results showed that depending on the type of vegetable, between 54% and 79% of the respondents were interested in iron-biofortified vegetables. Regression analysis showed a relationship between product acceptance, gender, and area of residence. In addition, relationships were found between consumer preferences for enjoyment, sustainability, and naturalness. Compared to functional food and dietary supplements, 77% of respondents would prefer fresh iron-rich vegetables to improve their iron intake. For a market launch, those iron-rich vegetables appear especially promising, which can additionally be advertised with claims for being rich in vitamin C and cultivated in an environmentally friendly way. Consumers were willing to pay EUR 0.10 to EUR 0.20 more for the iron-biofortified vegetables.

## 1. Introduction

Iron is an essential nutrient for the human organism that must be taken in regularly through the diet. It contributes to the maintenance of normal energy metabolism, the support of normal cognitive functions, the formation of red blood cells and hemoglobin, and the transport of oxygen in the body [1,2]. Around 2 billion people worldwide are affected by iron deficiency [3]. In Germany, the most recent nationally representative dietary survey showed that around 58% of women and 14% of men are affected by iron deficiency [4]. Women of childbearing age have an increased iron requirement due to blood loss during menstruation. The same applies to pregnant and breastfeeding women, who need additional iron to supply their fetus or infant [5,6]. Other risk groups for iron deficiency are individuals who are particularly active in sports and people whose diet is predominantly or exclusively composed of plant-based foods [7,8,9]. In athletes, the synthesis of hepcidin in the liver is stimulated by exercise, which reduces intestinal iron absorption [8,10]. Vegetarians and vegans have an increased risk of iron deficiency due to the lower bioavailability of plant-based iron [1,9]. Plant foods consist of trivalent iron, of which the body can absorb only about 1–15% from food. Animal foods, such as meat, poultry, and fish, contain divalent iron (heme iron). Humans can absorb 15–40% of this heme iron in their diet [1,9]. However, a variety of promoting and inhibiting substances are known to affect the uptake of non-heme iron. For example, some phytochemicals such as phytates and polyphenols, but also excessive amounts of zinc, magnesium, and manganese, inhibit the absorption of iron in the duodenum and proximal jejunum. In contrast, vitamin C, organic acids such as citric or lactic acid as well as the amino acids methionine and cysteine have an enhancing effect on this process. Accordingly, the composition of the diet is crucial for iron supply [9].

In addition to meat, functional foods and dietary supplements can also contribute to iron supply. Functional foods are foods that are fortified with nutrients during food processing to offer added health value [11]. Dietary supplements are nutrients in a tablet capsule or other dosage form [12]. Both product groups are often consumed more frequently by women than by men [13,14]. Around 20% of German women use iron supplements, and 21% use functional foods in any form [14].

However, iron sources of meat, dietary supplements, and functional food also have some disadvantages. Meat products do not reach the risk group of vegetarians and vegans, who represent 9% and 4% of the population in Germany, respectively [15]. In addition, high meat consumption increases the risk of cardiovascular disease, colorectal cancer, and type 2 diabetes [16]. High consumption of animal foods also seems to be detrimental from a sustainability point of view [17]. While the production of fresh vegetables generates an average emission of 153 g of carbon dioxide equivalents (CO_2_e) and frozen or canned vegetables between 415 to 511 g CO_2_e, meat generates between 3232 to 14,341 g of CO_2_e [18]. Food supplements and functional foods deter 39.8% of the German population, who value naturalness in food [19].

With this in mind, growing iron-rich vegetables with high iron bioavailability could be a healthy and sustainable option to improve people’s iron supply. In principle, various biofortification techniques can be used to increase the iron content of vegetables. These involve agronomic and breeding measures to enable vegetable crops to better absorb iron and transfer it to the edible parts of the plant. Although sufficient iron is usually present in soils, its phytoavailability is often limited. This can be countered by fertilizing with readily water-soluble iron chelates [20,21,22]. However, comparatively high iron fertilizer quantities are required for this. Foliar fertilization has proven to be more efficient for iron biofortification [23,24,25,26]. When vegetables are grown in soil-less cultivation systems, iron can also be supplied via the nutrient solution [27,28,29]. With regard to high bioavailability, it seems particularly desirable to biofortify those vegetables with iron that have a high vitamin C content, such as bell peppers, broccoli, kale, and arugula [30,31,32]. The selection and breeding of varieties that are naturally particularly rich in iron and vitamin C could further complement this [33,34]. Ideally, the genotypes should also contain comparatively low levels of substances that inhibit iron absorption in humans [35,36,37]. However, conventional breeding is a time-consuming undertaking [38,39]. Transgenic approaches, which have already been successfully tested in cereals such as rice, cannot be considered for use in countries of the European Union for legal reasons [40,41]. 

Fresh, iron-rich vegetables might be particularly appealing to people following a vegetarian or vegan diet. Women, who often consume more vegetables than men, are also likely to be attracted by such products [42]. In addition, consumers interested in a natural, healthy diet could be considered as a target group. Previous surveys in Europe on biofortified vegetables and fruits have shown that between 48 and 66% of the population are open-minded towards such products, especially those consumers who also value sustainability and naturalness [43,44,45]. At the same time, however, there is a high lack of knowledge about these products, which affects the purchase probability [43]. The aforementioned studies focused on fresh biofortified food with iodine, selenium, and lycopene. So far, very little information is available on consumer acceptance of iron-biofortified vegetables in Europe and other developed regions of the world. The situation is different for developing countries, where several biofortified products such as iron-rich beans and pearl millet are already widely used to combat hidden hunger [46,47,48,49]. However, the findings obtained there in accompanying consumer surveys cannot be transferred to the realities in industrialized countries because of the more serious malnutrition, the fundamentally different socio-economic conditions, and the divergent marketing channels for foodstuffs. 

Therefore, the aim of the present study was to examine the acceptance of German consumers towards iron-biofortified vegetables. The consumer survey conducted for this purpose addresses two main topic areas:

The first part deals with the characterization of the target group for iron-biofortified vegetables by means of the following research questions:

1. Does the acceptance of iron-biofortified vegetables depend on sociodemographic criteria as well as purchasing and consumption behavior?
2. Are there differences in consumer acceptance of vegetables biofortified with iron compared to vegetables enriched with other nutrients?
3. Do consumer groups that are attracted to iron-biofortified vegetables differ from those that consume functional foods and dietary supplements and, if so, in what respects?

The second part focuses on possible measures to increase acceptance and the potential willingness to pay for iron-biofortified vegetables. For this purpose, three research questions are addressed:

1. How do health claims and other labeled product attributes affect the likelihood of purchase?
2. Are potential buyers willing to pay a premium price for vegetables biofortified with iron?
3. What conclusion can be drawn from the general consumer behavior towards common vegetables for iron-biofortified vegetables?

## 2. Materials and Methods

### 2.1. Data Collection 

The data were collected in an anonymous quantitative online survey of German consumers (*n =* 1000). In January 2022, survey participants were recruited by Bilendi S.A., a licensed provider of online household panels for consumer research. For the consumer survey, a fully structured questionnaire was developed and implemented using the Tivian software from Tivian XI GmbH, Cologne, Germany.

The final questionnaire was preceded by a pretest that served to validate the items on purchase and consumption motives. A total of 225 participants took part in the pretest. The participants were acquired via various social media channels and are therefore not representative. 

### 2.2. Study Design

**Figure 1:**
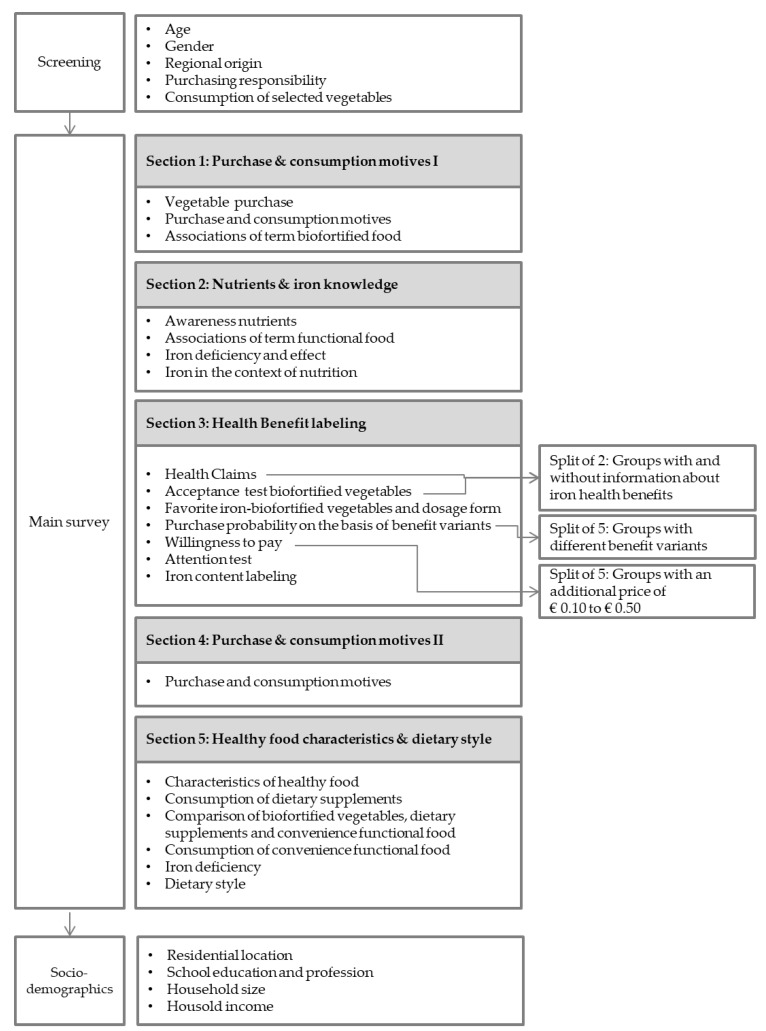
Graphical representation of the questionnaire design.

To answer the research questions outlined in the introduction, a questionnaire lasting approximately 20 min was designed with a total of 49 questions. The study focused primarily on five vegetables that are naturally high in vitamin C content: bell pepper, spinach, broccoli, arugula, and kohlrabi. Biofortifying such vegetables with iron is expected to result in high bioavailability of the trace element, as mentioned earlier. The breakdown of the questionnaire is shown in Figure 1.

The questionnaire began with a screening process that ensured that the participant sample was representative of age, gender, and place of residence of Germans. However, there are limitations to be noted with regard to representativeness. For example, only participants over the age of 18 who consume vegetables and are responsible for grocery shopping were surveyed. Slight deviations between the sample composition and the national average (Table 1) can be explained by screening with respect to the criteria of age and responsibility for grocery shopping. 

Section 1 of the main survey dealt with general purchasing and consumption behavior as well as specific purchasing criteria for vegetable selection. These questions served, among other things, to provide participants with an easy and familiar entry point to the survey. Furthermore, consumers were asked about awareness and associations of the term biofortified food. It is important to note that the term biofortified vegetables was used only in this context. Otherwise, the survey always referred to vegetables that contain more than twice the usual amount of iron due to special cultivation. Section 2 was specific to the nutrient iron. The aim was to be able to draw conclusions about the Germans’ level of knowledge about the nutrient. Furthermore, the associations with the term functional food were queried in order to be able to draw a comparison with the term biofortified food from the introductory part of the survey. Section 3 asked about the personal benefits of different health claims and the acceptance of iron-biofortified vegetables. To test whether information influenced the acceptance of iron-rich vegetables, participants were randomly divided into two groups (Group A and Group B). Group A received additional information about nutrient iron and its importance to health, while Group B received no information. After the acceptance question, all participants received the same questions again and were asked to select their preferred iron-rich vegetable. Five value-added claims were tested to determine the purchase probability of the five test vegetables compared to normal vegetables. For this purpose, participants were randomly assigned to groups A–E.

- Group A: Vegetable (preferred vegetable) is particularly rich in iron.
- Group B: Iron-rich (preferred vegetable) is also characterized by increased vitamin C content. Vitamin C promotes iron absorption in the body and thus supports an improved iron supply.
- Group C: The iron-rich (preferred vegetable) is also characterized by an increased content of iodine. Iodine is another vital trace element that contributes to normal thyroid function, among other things.
- Group D: Due to special cultivation, iron-rich (preferred vegetable) is less contaminated with undesirable substances such as cadmium and nitrate.
- Group E: Iron-rich (preferred vegetable) is less fertilized with nitrogen and has fewer crop losses. This results in less environmental impact and food losses in the production of the vegetable.

Participants were asked to indicate on a 0 to 10-point scale how likely they would purchase the biofortified vegetables compared to regular vegetables. Subsequently, this split was removed, and a new random 5-point split was conducted. This was carried out to be able to measure willingness to pay more across all labels. In the price test, participants were given a random additional price of EUR 0.10 to EUR 0.50 for their top 1 to 3 favorite vegetables compared to regular vegetables. Further, in this part of the survey, participants were asked about the popularity of different declaration options for iron content. Section 4 of the survey served to inquire about further general purchase and consumption motives. Section 5 focused on comparing iron-biofortified vegetables, iron supplements, and iron functional foods. Furthermore, the frequency of consumption of the latter two was surveyed. The survey was supplemented by the collection of additional sociodemographic data.

During data collection, incomplete questionnaires and interviews with very short completion times were excluded to ensure the quality of the data. In addition, interviews that did not pass the attention-level question were also excluded from the sample (Supplementary Material; Q34).

### 2.3. Statistical Analysis

Regression analysis was used to determine the relationships between the acceptability of iron-biofortified vegetables and sociodemographic, purchase, and consumption criteria. In addition, relationships between the latter criteria and consumption of functional foods and dietary supplements were determined and compared with iron-biofortified vegetables. 

For the linear regression of iron-biofortified vegetables, the acceptability query served as the basis. Since iron-biofortified vegetables cannot be consumed at present, no consumption frequency can be surveyed, only acceptance on a metric scale. For the dependent variable of the regression, the arithmetic mean was calculated across all test vegetables and Group A and B (groups with and without additional information about the importance of iron) (Supplementary Material; Q24, Q25). Demographic and sociodemographic characteristics and psychographic characteristics served as independent variables. In addition, purchase criteria were included. For the target group of functional foods and dietary supplements, an ordinal regression analysis was conducted with consumption frequency (dependent variable) and the independent variables mentioned above. An ordinal scale was used to interrogate consumption frequency. The subsequent comparison of the three regression analyses serves to show different measured values between the product groups' functional foods, e.g., iron-rich juice, iron dietary supplements, and iron-rich vegetables. In addition to the regression and the various descriptive analyses, cross-tabulation was used. 

All statistical analyses were performed using IBM SPSS Statistics 26 (IBM Corp., Armonk, NY, USA) and Microsoft Excel 2010 (Microsoft Corporation, Remond, WA, USA).

## 3. Results

### 3.1. Consumer Acceptance of Iron-Biofortified Vegetables Compared to Dietary Supplements and Functional Foods

Consumers of functional foods and dietary supplements were identified as potential target groups for iron-biofortified vegetables. However, the regression analyses also revealed differences in the characteristics of consumers of the three product categories (Table 2). 

Acceptance of iron-enriched vegetables was associated with female gender and urban residence. Individuals with a sustainable orientation and those who value the taste of food also showed increased interest in biofortified vegetables. The latter aspects were less relevant as a purchase motive for functional foods and dietary supplements. An exception in this context was the preference for products of local origin among consumers of functional foods. However, this relationship was relativized by the rather contrary result that this consumer group rarely visits farmers’ markets, organic food stores, or farm stores as food shopping locations. Functional foods tended to be preferred by younger individuals, while age was not a significant factor for the acceptance of iron-biofortified vegetables and the use of dietary supplements. Individuals using dietary supplements were more likely to be female, follow a special dietary style, and consume functional foods.

**Figure 2:**
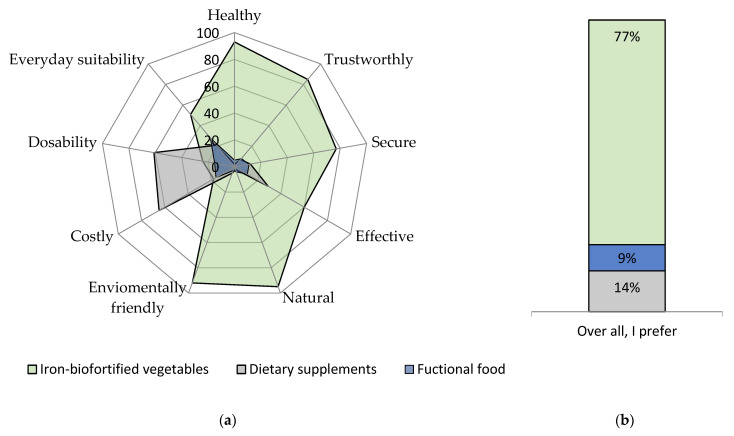
Consumer evaluation of iron-biofortified vegetables, iron-fortified functional foods, and iron-containing dietary supplements based on various criteria (**a**) and a comparison of preferences as a whole (**b**). Results refer to Q39 of the questionnaire, see Supplementary Materials. *n =* 1000.

For a direct comparison of the three product groups, survey participants were asked to rate a selected iron-biofortified vegetable with an iron-fortified fruit juice drink and an iron-containing dietary supplement on various criteria (see Supplementary Material, Q33 and Q39 for product images shown). Nine out of ten participants rated the iron-biofortified vegetables as particularly natural, healthy, and sustainable compared to the other two product groups (Figure 2a). The majority of consumers also rated iron-biofortified vegetables as superior to iron-containing functional foods and dietary supplements in terms of safety, efficacy, and suitability for everyday use. In contrast, relatively few consumers were convinced of the good dosing properties of the iron-biofortified vegetables, while six out of ten participants attributed this trait to dietary supplements. Functional foods convinced respondents only in terms of affordability. Overall, 77% of the consumers would prefer iron-biofortified vegetables to improve their iron supply. Only 14% favor dietary supplements and 9% functional foods for this purpose (Figure 2b).

### 3.2. Consumer Knowledge of Iron Deficiency Disorders and Sources of Iron in the Diet

**Figure 3:**
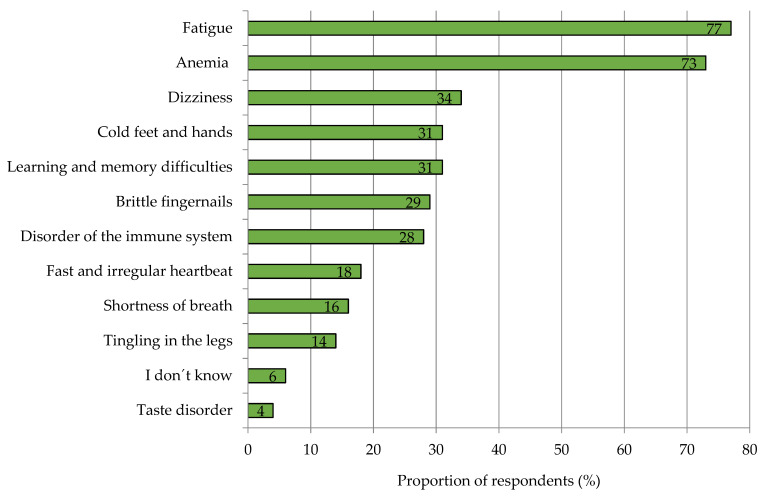
Awareness of iron deficiency symptoms among respondents. Results refer to Q18 of the questionnaire, see Supplementary Materials. *n =* 1000.

**Figure A1:**
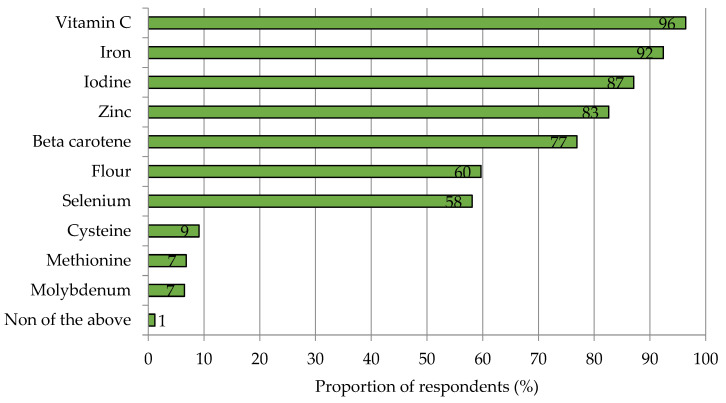
Awareness of nutrients that German consumers have heard in connection with nutrition. Results refer to Q13 of the questionnaire, see Supplementary Materials. *n =* 1000.

Among the German consumers surveyed, vitamin C proved to be the best-known nutrient, with a level of awareness of 96%. Iron was known by 92% of the respondents, and iodine followed in third place with 87% (Appendix A, Figure A1). With regard to the health effect of iron on the human organism, four out of five participants stated that iron had a positive effect. Only 15% were unsure, and 1% assessed the effect as rather negative. Along with the high level of awareness, over 70% of participants correctly stated that fatigue and anemia were among the symptoms of iron deficiency (Figure 3). Other symptoms of iron deficiency were significantly less known. Only about two out of six could attribute the symptoms of dizziness, cold feet and hands, learning and memory difficulties, brittle fingernails, and immune system disorders to iron deficiency.

**Figure A2:**
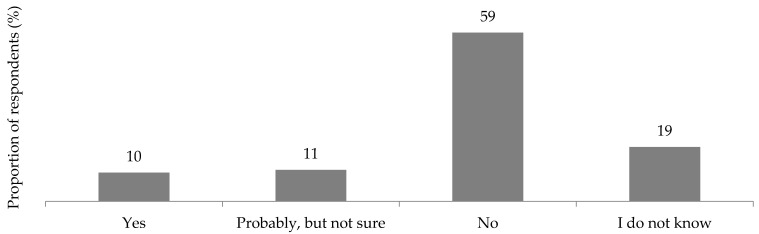
Survey participants who are iron-deficient. Results refer to Q41 of the questionnaire, see Supplementary Materials. *n =* 1000.

**Figure A3:**
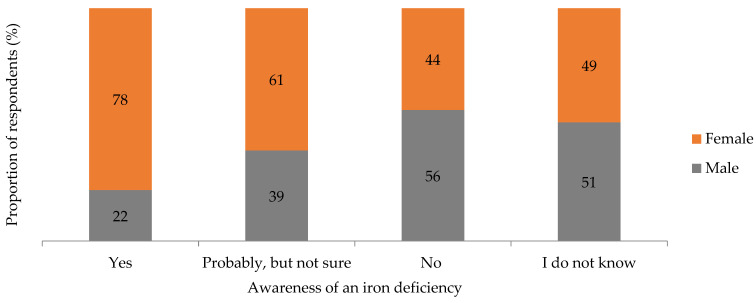
Germans who are temporarily affected by iron deficiency in their immediate family. Results refer to Q2 and Q41 of the questionnaire, see Supplementary Materials. *n =* 1000.

In the survey, 10% of the participants reported having an iron deficiency. Of these, 78.1% were women and 21.9% were men. A total of 60% of respondents believed that iron deficiency did not affect them, and 30% were not sure in this regard (Appendix A, Figure A2 and Figure A3).

**Figure 4:**
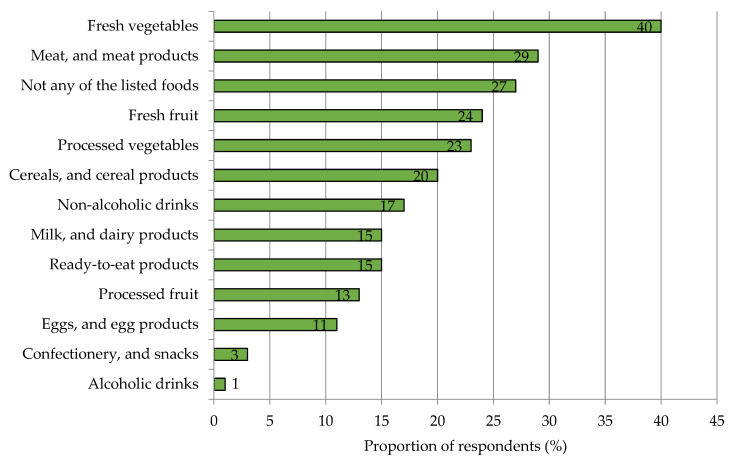
Food groups considered appealing by respondents for iron enrichment to meet personal iron requirements (multiple choice possible). Results refer to Q22 of the questionnaire, see Supplementary Materials. *n =* 1000.

**Figure A4:**
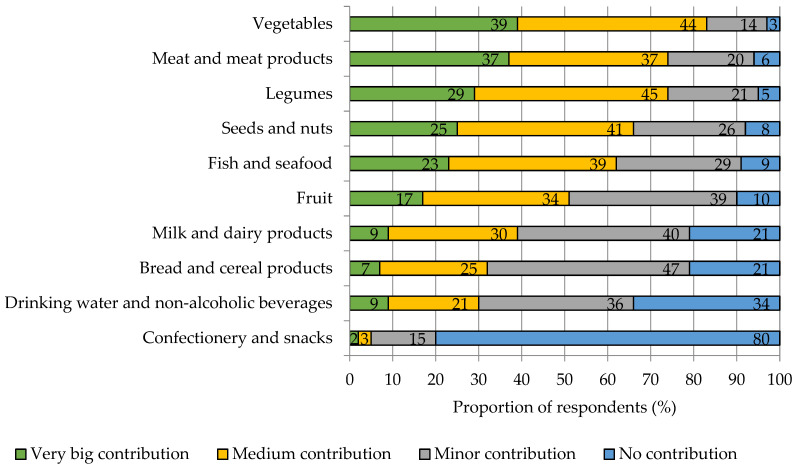
German consumers’ assessment of the personal benefits of various foods for iron supply. Results refer to Q20 of the questionnaire, see Supplementary Materials. *n =* 1000.

Two out of five participants assume that vegetables make a very large contribution to their personal iron supply. They were followed with a small margin in the rating by meat and meat products (37%), legumes (29%), seeds and nuts (25%), and fish and seafood (23%) (Appendix A, Figure A4). When respondents were asked which food groups they perceived to be most appealing to increase iron content for improving their supply with the micronutrient, fresh and processed vegetables were also the most frequently mentioned vegetables (63%). Fresh and processed fruits were the second most popular choice (37%), and meat and meat products ranked third (29%) (Figure 4). A total of 27% of respondents expressed a fundamental opposition to foods with increased iron content.

**Figure 5:**
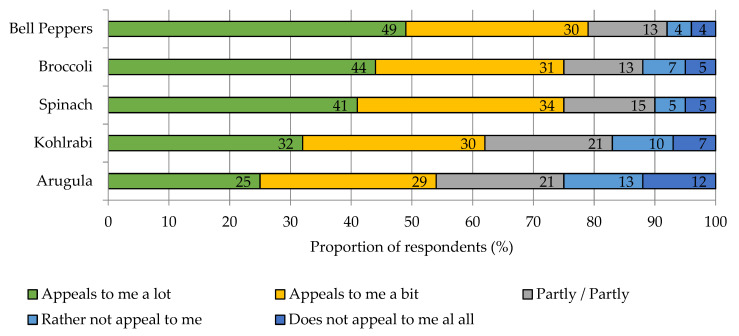
Acceptance toward selected iron-biofortified vegetables with high native Vitamin C content. Results refer to Q24 and Q25 of the questionnaire, see Supplementary Materials. *n =* 1000.

Among the five iron-biofortified and vitamin C-rich vegetables used as examples in this survey, consumer acceptance ranged from 54% to 79%. Acceptance was lowest for arugula and highest for bell peppers (Figure 5).

### 3.3. Factors Relevant to the Market Introduction of Iron-Biofortified Vegetables in Germany

**Figure 6:**
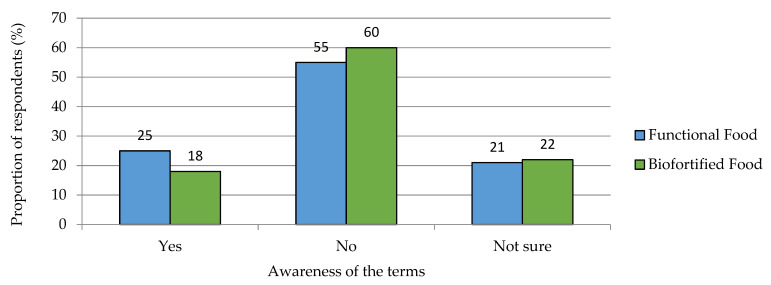
Awareness of the terms functional foods and biofortified foods among respondents. Results refer to Q10 and Q14 of the questionnaire, see Supplementary Materials. *n =* 1000.

Only a relatively small proportion of respondents were familiar with the terms “functional foods” and “biofortified foods” (Figure 6).

**Figure A5:**
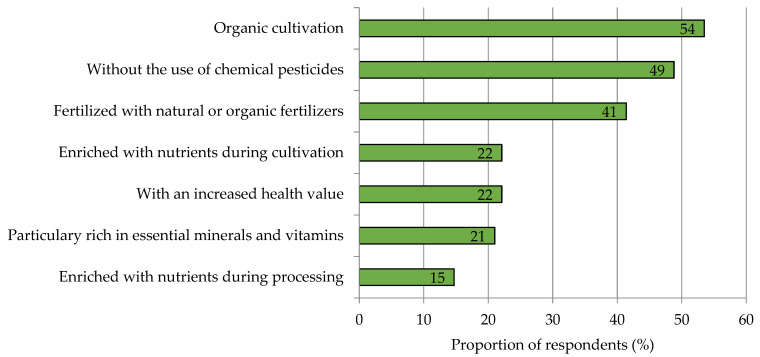
Characteristics that German consumers attribute to biofortified foods. Results refer to Q11 of the questionnaire, see Supplementary Materials. *n =* 1000.

**Figure A6:**
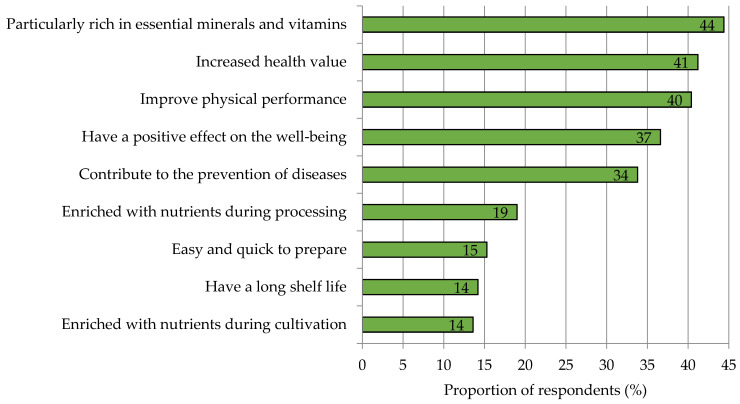
Properties that German consumers attribute to functional foods. Results refer to Q15 of the questionnaire, see Supplementary Materials. *n =* 1000.

When asked what characteristics participants associate with the term functional food, 44% indicated foods “particularly rich in essential minerals and vitamins”. Around four out of ten respondents viewed these foods as having “increased health value” or as “improving physical performance”. Thus, they intuitively associated the right properties with the products despite great unfamiliarity with the term. The term biofortified foods was understood by more than half of the participants to mean “products from organic farming”. In addition, 49% associated it with “foods produced without the use of chemical pesticides” and 41% with “foods fertilized with natural or organic fertilizers”. Only one in five respondents was able to correctly match the term, associating it with foods that have been enriched with nutrients during cultivation and thus provide additional health benefits (Appendix A, Figure A5 and Figure A6).

**Figure 7:**
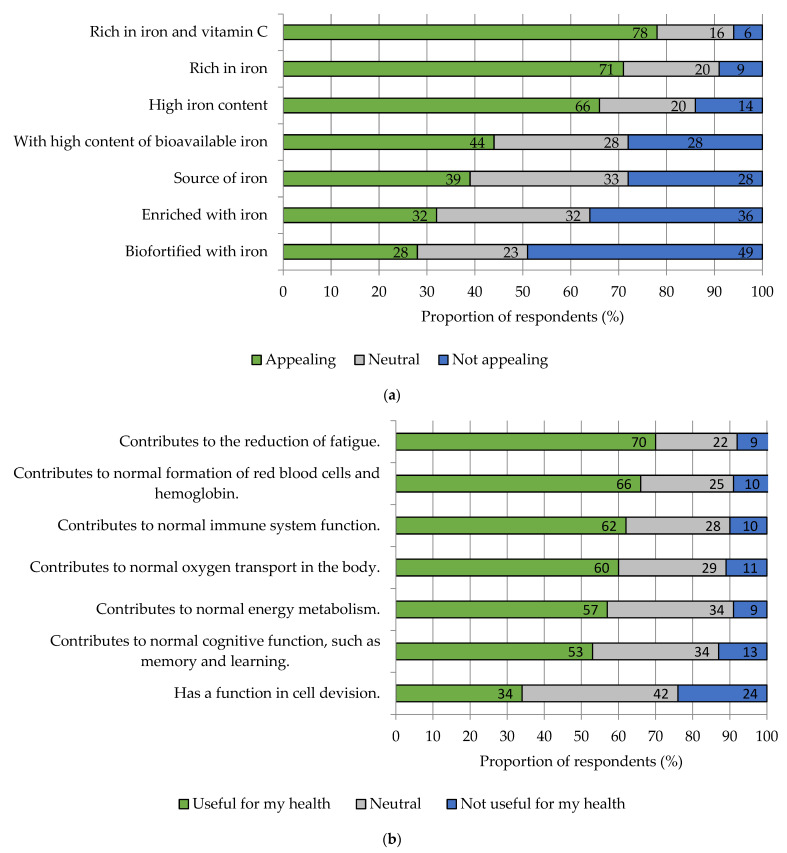
Attractiveness of nutrition claims (**a**) and health claims (**b**) for iron-rich vegetables in the purchase decision for iron-rich vegetables. Results refer to Q23, Q26, and Q35 of the questionnaire, see Supplementary Materials. *n =* 1000.

Iron-biofortified vegetables may be labeled with certain health claims if they achieve the minimum legally regulated levels of iron. German consumers are most likely to be attracted by the claim “rich in iron” (71%) and “rich in iron and vitamin C” (78%) (Figure 7a). Thus, the survey results show that the combination of iron with a well-known vitamin increases the acceptance of iron-biofortified vegetables. The label “biofortified with iron” scored the lowest (28%). Among the health claims, the aspect “helps to reduce fatigue and tiredness” (70%) scored the highest (Figure 7b). It is striking that six of the seven health claims differed only slightly in their ratings. Their “useful for my health” rating varied between 53% and 70%. Only the claim “has a function in cell division” scored significantly worse, with 34% agreement on usefulness for one’s own health.

**Figure A7:**
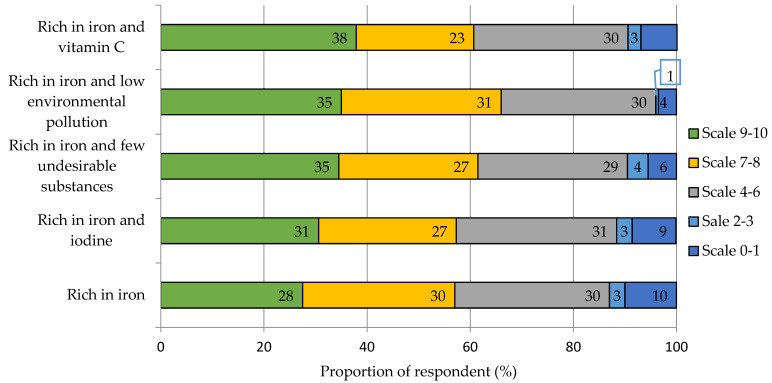
Purchase likelihood of iron-biofortified vegetables by health claims in percent. Scale 0 = would buy normal vegetables; 10 = would buy iron-rich vegetables. Results refer to Q32 of the questionnaire, see Supplementary Materials. *n =* 200.

The purchase probability of vegetables biofortified with iron was investigated as a function of various advertising claims. Each group was shown a label and asked to rate the purchase probability of iron-biofortified vegetables compared to normal vegetables on a scale of zero to ten. Focusing on the 9–10 score as clearly interested in purchasing, 28% of consumers would definitely buy a biofortified vegetable labeled “rich in iron” (Appendix A, Figure A7). The combined declaration with the trace element iodine did not achieve significantly higher purchase probability. The indications “few undesirable substances” and “low environmental impact” increased the purchase probability by about 7%. The combination of rich in iron and vitamin C increased the purchase probability by 10%. 

The purchase probability of iron-biofortified vegetables varied among the different target groups depending on the labeling of the product (Table 3). Half of the vegans surveyed indicated a high purchase probability for the labels “rich in iron and iodine” and “rich in iron and low environmental impact”. Vegetarians felt attracted to purchase by the labels “rich in iron and low environmental impact” (53%) and “rich in iron and low undesirable substances” (46%). Among women, a label combination generally increased the likelihood of purchasing. For men, the probability of purchase decreased by 8% when the label “rich in iron and iodine” was used, compared to only “rich in iron”. Furthermore, the comparison shows differences in label preference depending on the place of residence and the sustainability and purchasing behavior. While the labels “rich in iron and few undesirable substances” and “rich in iron and low environmental impact” are preferred in cities and regions near cities, additional claims with vitamin C or iodine are favored in rural areas. Highlighting extra vitamin C was also appreciated by consumers who particularly value taste in their food selection. Likewise, this was observed among consumers with a particularly sustainable attitude. Surprisingly, the indication of environmentally friendly cultivation had less of an effect on the likelihood of purchase among this group of consumers. The environmental claim had the highest purchase probability among consumers who prioritize plastic-free packaging. 

**Figure 8:**
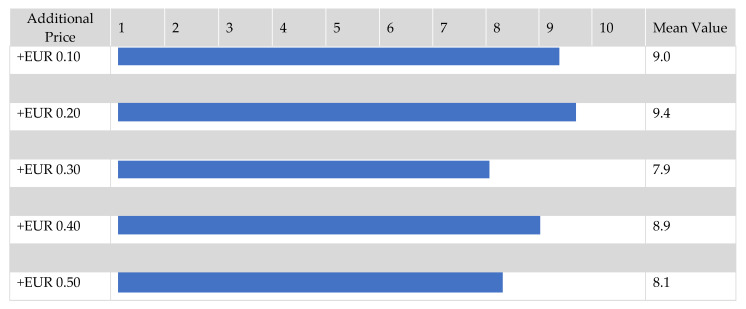
Purchase probability of iron-rich vegetables as a function of additional price compared to “normal” vegetables. Results refer to Q33 of the questionnaire, see Supplementary Material. (*n =* 218). Queried on a 10-point scale. Participants were randomly assigned to price groups in a 5-way split.

Respondents who indicated a high likelihood of purchasing iron-biofortified vegetables (21.8% of the sample) also expressed a willingness to pay a premium price. Across all five exemplary iron-biofortified vegetables (bell pepper, broccoli, spinach, arugula, and kohlrabi), an additional willingness to pay of EUR 0.10 to EUR 0.20 was found (mean value ≥9.0 on a 10-point scale, Figure 8). For a 250 g bag of spinach, this corresponded to an additional willingness to pay of 5.6–11.2%. For a 125 g bag of arugula, it was 11.2–22.5%; for a head of broccoli, 8.4–16.8%; for a set of 3 bell peppers, 9.2–18.4%; and for a kohlrabi, 20.4–40.8% (product images in Supplementary Material, Q33). At a premium of EUR 0.30, the purchase probability decreased. Nevertheless, the purchase probability still ranged from 7.3 to 8.9 at an additional price of EUR 0.30 to EUR 0.50.

### 3.4. Food Preferences and Purchasing and Consumption Behavior of Germans

**Figure A8:**
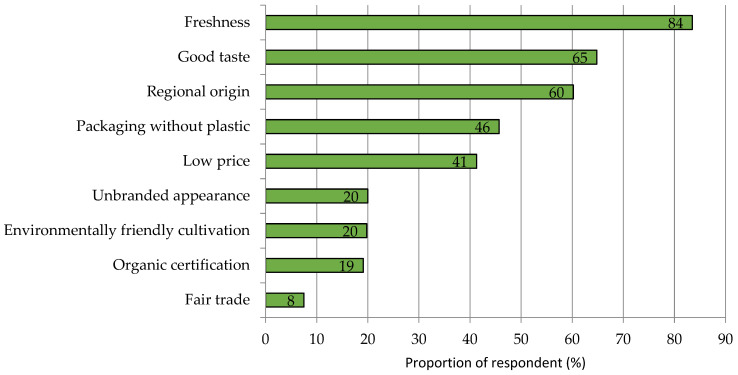
Important aspects when buying fresh vegetables. Participants could select up to four criteria. Results refer to Q8 of the questionnaire, see Supplementary Materials. *n =* 1000.

**Figure A9:**
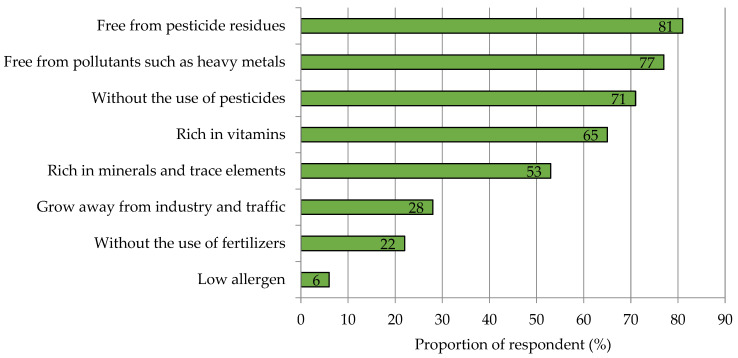
Important properties that characterize healthy vegetables based on the participant’s favorite vegetable. Multiple Choice. Results refer to Q37 of the questionnaire, see Supplementary Materials. *n =* 1000.

When buying vegetables, German consumers pay particular attention to freshness, taste, and regional origin. For more than one in two, these are important purchasing criteria. Around 46% of respondents pay attention to plastic-free packaging when buying vegetables. Organic labels (19%) and fair-trade labels (8%) receive less attention (Appendix A, Figure A8). By the term “healthy vegetables” German consumers understand the vegetables to be “free from pesticide residues” (81%), “free from pollutants such as heavy metals” (77%), and “without the use of pesticides” (71%). More than half of the consumers surveyed associated characteristics such as “rich in vitamins “ and “rich in minerals and trace elements” with the term healthy vegetables (Appendix A, Figure A9).

**Figure A10:**
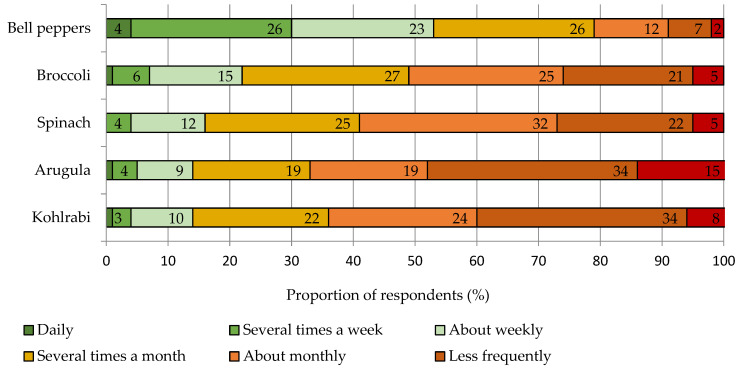
Consumption frequency of selected vegetable products. Results refer to Q5 of the questionnaire, see Supplementary Materials. *n =* 1000.

**Figure A11:**
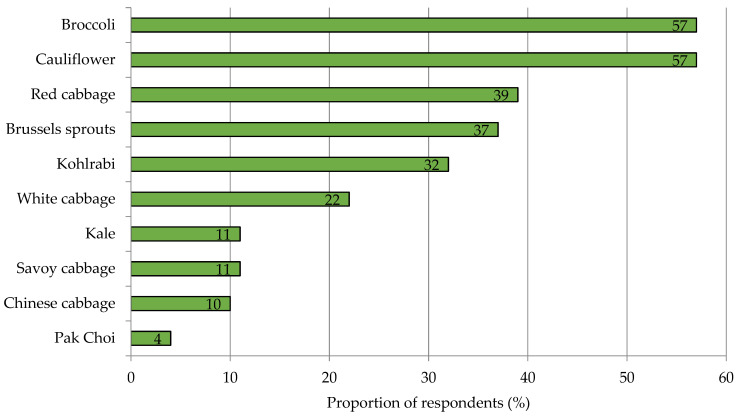
Most popular types of cabbage in Germany by frequency of consumption. Results refer to Q6 of the questionnaire, see Supplementary Materials. *n =* 1000.

Consumers were shown five vegetables that are naturally high in vitamin C. Bell pepper was among the most frequently consumed vegetables, followed by broccoli and spinach. Arugula and kohlrabi were consumed infrequently, mostly once a month or less (Appendix A, Figure A10). In addition, the frequency of consumption of various types of cabbage vegetables was queried. Again, broccoli was found to be among the most popular cabbage vegetables. Cauliflower, red cabbage, and brussel sprouts were also frequently consumed (Appendix A, Figure A11).

**Figure A12:**
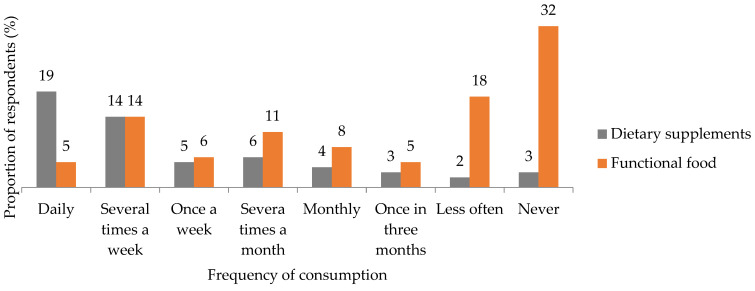
Frequency of consumption of dietary supplements and functional food in Germany. Results refer to Q38 and Q40 of the questionnaire, see Supplementary Materials. *n =* 1000.

Dietary supplements and functional foods represent existing options for improving individual nutrient supply. A total of 19% of the survey participants stated that they consumed dietary supplements such as iron, magnesium, or vitamin C preparations on a daily basis. One in five consumers consumes these products once or several times a week. Almost half of respondents stated that they rarely or never use dietary supplements. Functional foods such as fruit juices with added vitamin C, probiotic yogurt, bread with omega-3 fatty acids, or energy drinks containing caffeine were used by few survey participants. Only 5% said they consumed these products daily, and 21% once or several times a week. Again, about half say they rarely or never used functional food (Appendix A, Figure A12).

## 4. Discussion

### 4.1. Comparing Consumer Groups for Iodine-Biofortified Vegetables with Those for Functional Foods and Dietary Supplements 

The survey of German consumers revealed a higher acceptance of iron-biofortified vegetables among women and urban residents. This is likely due to the fact that women are more likely to suffer from iron deficiency and are therefore more aware of the health consequences (Appendix A, Figure A3) [3,4]. Urban areas are home to a larger proportion of vegetarians and vegans, who are at greater risk of iron deficiency [7,14]. Additionally, urban consumers may be more willing to experiment with new foods compared to their rural counterparts [14]. In contrast to previous studies on biofortified foods, in the present case, no association was found between the acceptance of biofortified vegetables and age [51,52]. This may be due to the fact that iron deficiency is relevant in almost all age groups due to the high number of affected people [3,4,8]. Increased acceptance of iron-biofortified vegetables was significantly associated with sustainability awareness in terms of purchasing and consumption criteria. The importance of sustainability and naturalness in interaction with the acceptance of biofortified vegetables and fruits was already shown in previous studies [43,44,45]. The taste of vegetables proved to be an important purchasing criterion, as likewise reported for other foods, including functional foods. Consumers’ willingness to compromise on taste for the sake of their health is limited [53,54]. Hence, it is essential to take special care during the production of biofortified vegetables to preserve their taste quality and prevent any adverse effects on flavor. 

Unlike to iron-biofortified vegetables, in the case of functional foods, there was a slight trend toward preference by younger consumers, consistent with previous reports [14,15,52]. However, opposite relationships are also reported in the literature [51,54]. Also differing from previous reports, lower acceptance of functional foods was found among women compared with men [14,54]. The different results on the use of functional food can be explained by the fact that there are a large number of functional food products, e.g., vitamin-enriched, probiotic, and fat-reduced, addressing various target groups with diverse consumption motives [55,56].

Interestingly, buyers of functional foods also attach importance to the regional origin of the foods they buy. This may be related to the fact that regionality has become more important in times of COVID-19 [57]. Apparently in contradiction to this is the fact that functional food users tend to buy less in organic stores, farm stores, and weekly markets. Here, however, it can be assumed that these shopping locations have a small product portfolio of convenience products and thus do not represent a common shopping location for this consumer group. The significant relation between the frequency of use of functional foods and dietary supplements is likely to be explained by the health focus of both user groups [43,58].

Users of dietary supplements were characterized by a tendency to follow special diets such as veganism or a lower-carb, protein-rich, or pescetarian diet. Likewise, in previous studies, dietary supplement users were characterized by a healthier lifestyle. This was characterized by a better diet, expressed by a higher intake of nutrients, physical activity, and weight control [58]. Further, users are characterized by their female gender, as previous studies have also shown [58,59]. Users of dietary supplements often also consume functional foods. This confirms the overlap between the two health-oriented user groups mentioned earlier.

The significant correlation between functional food consumption, supplement consumption, and the acceptance of biofortified foods suggests that consumers rarely differentiate between these product categories but rather make purchasing decisions based on their individual dietary and health needs.

### 4.2. Consumers’ Level of Knowledge about Micronutrient Iron

Iron was the second best-known nutrient in the diet (92%). Only vitamin C was even better known, at 96%. Recent surveys have shown that between 2020 and 2021 in Germany, the number of iron supplement users increased by 4.0%, and general interest in health products increased by 2.2% [14]. This development in nutrient awareness could be related to the increased health awareness during the COVID-19 pandemic. During this time, people were increasingly confronted with the topics of health and nutrition [60].

Awareness of iron as a nutrient was high in the survey, but knowledge of iron deficiency diseases was limited. The two best-known deficiency symptoms, fatigue and anemia, were known to over 70% of the participants. The nine further queried symptoms were nearly unknown. This lack of knowledge fits with the other findings of this study that only 10% of respondents reported suffering from iron deficiency. According to earlier studies, the number of people affected in Germany is much higher, at 58% for women and 14% for men [4]. However, the discrepancies may be explained by the fact that many people do not report the often unspecific symptoms to their general practitioner, as they are partly seen as a consequence of the aging process [61]. In order to sensitize the population to a possible iron deficiency, it is therefore necessary to raise awareness of the other deficiency symptoms. This could be carried out by means of a large-scale educational campaign, extending to social media, aimed in particular at risk groups. In this context, consumer knowledge of iron sources should also be trained because here, too, the study showed discrepancies between the actual and estimated sources of iron in the diet. For example, consumers ranked vegetables as the most important personal source of iron, followed by meat and meat products, legumes, and seeds and nuts. However, vegetables usually have a low iron content [62]. 

The finding that consumers suspect vegetables as an important source of iron in their diets is likely to be favorable for the acceptance of iron-biofortified products in this food category. In fact, respondents also most often chose fresh vegetables when asked which food group was considered appealing for iron enrichment to improve their personal iron supply. Processed vegetables as well as fruits in fresh and processed forms were also relatively popular in this regard (Figure 4). Similar results were observed in previous studies on iodine- and selenium-biofortified foods [45,63]. In the current survey, interest in iron-rich fresh vegetables increased from initially 40% up to 79% when shown a specific product preferred by consumers with appropriate labeling. This relatively high level of interest is likely to be due to the fact that iron is better known to consumers and tends to be rated more positively in terms of its health effects compared with iodine and selenium [45,63]. Accordingly, the study indicates a substantial demand potential for iron-biofortified vegetables.

### 4.3. Impact of Consumer Perceptions on Nutrient- and Health-Related Terms and Claims for a Market Launch of Iron-Biofortified Vegetables

Only a small proportion of the German consumers surveyed are familiar with the term “biofortified food” (18%). This result was to be expected, since on the one hand, the offer of biofortified foods in German food retail stores is still very low. The few examples include a selenium-rich apple and vitamin D-rich mushrooms [64,65]. On the other hand, the marketing of these products does not refer to biofortification. This results from legal requirements of REGULATION (EU) 2018/848, according to which the term “bio” is allowed only for products from organic cultivation [66]. The risk of confusion of the word “bio” is confirmed by the results of this survey. About half of the respondents associated biofortification with food from organic cultivation, grown without chemical pesticides. Only about two out of ten respondents correctly associated the term with its intended meaning (Appendix A, Figure A5). Even for nutrient-enriched organic vegetables, the use of the term “biofortification” in marketing is not useful because it is considered unattractive by consumers (Figure 7a). Surprisingly, the term “functional food” is not much better known in Germany, although around one-third of Germans consume these products at least occasionally [67]. The reason for this is probably that the expression is hardly used in product claims. Instead, specific product attributes and health claims are used for marketing. Despite the low awareness of the term functional food, four out of ten associated the correct content meaning (Appendix A, Figure A6). 

Appropriate nutritional labels can be legally used if the required iron content is achieved. The label “rich in iron and vitamin C” not only achieved high acceptance in the survey but also led to a 10% increase in the overall purchase probability compared to “rich in iron”. This result supports the hypothesis mentioned above that nutrient awareness increases the acceptance of biofortified foods. The inclusion of “vitamin C” in the claim had a particularly strong effect on the willingness to purchase among consumers who follow a specific dietary pattern (e.g., people on low-allergen diets, flexitarians) and those who value environmentally friendly farming practices (Table 3).

In addition to nutritional declarations, legally authorized health claims can also support the communication of iron-biofortified foods [68,69]. Here, the claim “contributes to the reduction of fatigue and tiredness” convinced 70% of participants, followed by the health claim “contributes to normal formation of red blood cells and hemoglobin” with 66%. Comparing the best-known iron deficiency diseases with respondents’ preferred health claims reveals overlap. The rather general health claim “supports the normal function of the immune system”, which found the highest approval in previous consumer studies [63], followed in third place. The apparently increasing preference for specific health claims may be due to increasing health awareness triggered by the COVID-19 pandemic, which led to a more differentiated view of nutrition, nutrients, and health effects among consumers [57].

Along with purchase probability, willingness to pay plays an important role in the market launch of this new product group. Individuals with a high interest in iron-biofortified vegetables would mostly accept a price premium of EUR 0.10 to EUR 0.20. Thus, the willingness to pay more was 23% across all vegetable varieties. This is in line with the data from a previous meta-analysis, where this figure ranged from 20 to 25% [48]. However, in this study, even with the highest price supplement of EUR 0.50, only a relatively small decrease in purchase probability was observed (Figure 8). Accordingly, it can be assumed that the customers are not very price-sensitive. Foti et al. attribute the low influence of price on the purchase decision for functional products to the nutritional awareness of buyers [70].

A look at the general purchasing and consumption behavior of vegetables shows relevant criteria that should be taken into account when introducing iron-rich vegetables into the market. As in previous studies [45,63], the supermarket proved to be the most important place for purchasing fresh vegetables (results not shown). Freshness plays the most important role in product selection. Good taste as well as a regional origin and “freedom from” are other key criteria (Appendix A, Figure A8) [19,53]. In order for consumers to obtain an impression of the taste, tastings should be conducted at the point of sale when the product is introduced in the market. In addition, the origin of the vegetables as well as the “free from” information should be indicated on the packaging. At the same time, the packaging of the vegetables should be plastic-free. 

As this study showed, bell peppers followed by broccoli and spinach are among the most commonly consumed vitamin C-rich vegetables (Appendix A, Figure A10 and Figure A11). Furthermore, they are also the most popular among consumers when iron-biofortified (Figure 4). Accordingly, the development of vegetables with high bioavailable iron content should focus on these product groups as a first step.

The motives for purchasing and consuming biofortified vegetables differ from those for functional foods and dietary supplements. Here, sustainability and health in combination with taste play a significant role. Corresponding added values should be taken into account in packaging design. Compared to the other two product groups, consumers rated iron-enriched vegetables as a particularly natural way to improve iron supply, as already found in previous surveys with biofortified fresh products [45]. However, more information about the positive effect on the human organism, suitability for everyday use, and dosage was desired by the respondents. This needs to be addressed on the packaging, e.g., in the form of nutrient declarations, health claims, and consumption recommendations. To meet these consumer information expectations, recognizable product labeling across different nutrients, food groups, and processing stages should be the aim [43].

### 4.4. Limitations of the Study and Future Research Potentials

This study investigated German consumers’ attitudes toward iron-biofortified vegetables. For a better understanding, five types of vegetables were used as specific examples in the survey. The selected vegetables are characterized by an inherently high vitamin C content as well as frequent consumption by consumers. This is also a limitation of the study. Only a limited number of vegetables possible for iron biofortification could be shown. Fruit products were also not considered. The same applies to the further processing of iron-biofortified vegetables. This was also only slightly addressed in the survey. Future studies should therefore include other vegetable and fruit varieties suitable for iron biofortification. In addition, the potential for further processing in the form of frozen products, vegetable mixes, and convenience products should be investigated. This could take the form of a discrete choice experiment while also considering design and labeling aspects relevant to market introduction. A further limitation of the study lies in the additional price query. Here, the actual willingness to pay more should be determined by a real experiment at the point of sale once the iron-rich vegetables have been developed. In this way, the actual purchasing behavior as well as the advertising impact of different designs and labeling could be analyzed and related to the willingness to pay a higher price. Moreover, for a final target group determination, the regression analysis should be supplemented by a cluster analysis. In addition to the limitations and the resulting further research opportunities, the study showed the possible potential of uniform biofortification labeling across different product groups. Labeling with an accompanying information campaign could significantly increase the uptake of iron-biofortified foods if the experts and certification bodies are trustworthy and well known [47,70,71]. 

## 5. Conclusions

Iron-biofortified vegetables are proposed as a new approach to enhance iron intake in individuals at increased risk for iron deficiency, such as women, vegetarians, vegans, and athletes. When selecting vegetables suitable for this purpose, attention should be paid to a naturally high vitamin C content to ensure high iron bioavailability. Such plant-based products could be an adequate supplement to current iron sources such as meat, functional foods, and dietary supplements. Many German consumers would prefer the new biofortified vegetables not only to functional foods or dietary supplements (77%) but also to regular vegetables (63%). The preference for iron-rich vegetables can be increased by combining them with another characteristic such as “rich in vitamin C” or “environmentally friendly cultivation”. First-time buyers of iron-biofortified vegetables are particularly likely to be people living in urban areas who value taste, sustainability, and naturalness. When launching the products, it is important to communicate this added product value both in the packaging design and at the point of sale. The market launch is ideally supported by an information campaign and cross-product ingredient labeling that addresses consumer-relevant issues such as contribution to iron intake, efficacy, and suitability for everyday use. The effectiveness of such measures would be enhanced by support from policy makers and trusted public institutions such as nutrition societies and medical associations. In addition, a legal framework that clearly defines the characteristics and requirements for biofortified products would be helpful in increasing acceptance and credibility among consumers. Potential customers for iron-biofortified vegetables are willing to pay an average price premium of 23% compared to corresponding common goods. This creates new opportunities for product portfolio diversification in the fresh produce segment. Overall, this product innovation offers promising prospects for growers and retailers looking to expand their product range and meet the changing needs and preferences of health-conscious consumers. If successfully launched on the market, iron-biofortified vegetables can contribute to a sustainable improvement in dietary iron supply.

## Figures and Tables

**Table 1:** Attributes of the consumer sample (*n =* 1000) compared to the national average.

Characteristics		Test Sample (%)	Population Level Germany 2021 (%) [14,50]
Consumption of fresh fruit and vegetables	Consume at least rarely	100	85
Gender	Male	50	51
	Female	50	49
Age	18–24	9	7
	25–34	15	13
	35–44	15	13
	45–54	17	13
	55–64	18	15
	>65	26	22
Region in Germany	North	18	18
	West	35	35
	South	29	29
	East	18	18
Responsible for purchasing	Mainly myself	64	63
	Myself and another person	36	35

**Table 2:** Relationships between acceptance of iron-biofortified vegetables, functional foods, or dietary supplements and various consumer characteristics. *n =* 1000.

	Iron-Biofortified Vegetable R^2^ = 0.077	Functional Food R^2^ = 0.118	Dietary Supplement R^2^ = 0.144
Dietary Style			
No special	−0.081 * (−2.498)	−0.015 (0.013)	−0.717 ** (31.285)
Shopping location			
Farmer’s market, organic produce shop, farm store	0.045 (1.440)	−0.503 ** (6.941)	0.165 (0.699)
Sociodemographic variable			
Age	0.062 (1.896)	−0.025 ** (39.322)	0.006 (2.246)
Female Gender	0.078 * (2.430)	−0.330 ** (6.913)	0.384 ** (8.924)
Rural Area	−0.078 * (−2.548)	−0.084 (1.229)	−0.109 (1.937)
Sustainability awareness			
Environmentally friendly cultivation	0.105 ** (3.332)	0.199 (1.642)	0.185 (1.317)
Regional origin	0.079 * (2.488)	0.338 ** (7.043)	−0.110 (0.709)
Plastic-free packaging	0.068 * (2.155)	0.055 (0.199)	−0.154 (1.446)
Purchase criteria			
Good taste	0.062 * (1.962)	0.111 (0.745)	0.041 (0.094)
Sources of iron supply			
Functional Food	0.134 ** (4.171)	−/−	0.239 ** (78.493)
Dietary Supplements	0.068 * (2.101)	0.161 ** (55.115)	−/−

The table shows the results of linear and ordinal regression analyses. Linear regression was used for the iron-biofortified vegetables. The first value per column indicates the standardized beta coefficient; the asterisks symbolize the significance level: ** = α < 0.01 highly significant; * = α < 0.05 significant. The values in brackets indicate the t-value. Ordinal regression was used for functional food and dietary supplements. The first value per column indicates the estimator; the asterisks symbolize the significance level: ** = α < 0.01 highly significant; * = α < 0.05 significant. The values in brackets represent the Wald value.

**Table 3:** Purchase probability of iron-rich vegetables as a function of labeling from the perspective of different consumer groups.

	Rich in Iron	Rich in Iron and Vitamin C	Rich in Iron and Iodine	Rich in Iron and Few Undesirable Substances	Rich in Iron and Low Environmental Impact
Dietary style					
Flexitarian	26.8%	43.2%	42.9%	31.9%	46.1%
Vegan	22.2%	33.3%	50.0%	42.9%	50.0%
Vegetarian	33.4%	28.6%	21.4%	46.1%	53.4%
Low allergen diet	33.0%	50.0%	16.7%	66.7%	37.5%
No special diet	26.9%	34.3%	25.0%	34.1%	24.3%
Gender					
Female	24.7%	41.6%	38.0%	32.6%	37.2%
Male	30.1%	34.0%	22.0%	36.2%	32.6%
Rural area					
City center	34.9%	33.9%	19.7%	45.0%	29.6%
City outskirts	26.1%	38.1%	36.1%	32.9%	40.9%
Rural	22.1%	40.5%	34.9%	26.5%	31.5%
Sustainability awareness					
Environmentally friendly cultivation	25.0%	54.8%	45.9%	36.9%	35.6%
Regional origin	25.0%	41.2%	35.1%	38.0%	35.1%
Plastic-free packaging	26.2%	36.3%	30.9%	33.0%	39.8%
Purchase criteria					
Good taste	27.0%	35.3%	25.7%	29.7%	30.3%

## Data Availability

The data presented in this study are available on request from the corresponding author. The data will be made public at a later stage.

## Supplementary Materials

The supporting information can be downloaded at: https://www.mdpi.com/article/10.3390/nu15102291/s1.
