# Supplementary material for: Vegetables with Enhanced Iron Bioavailability—German Consumers’ Perceptions of a New Approach to Improve Dietary Iron Supply
Source: Nutrients. 2023 May 12;15(10):2291. doi: 10.3390/nu15102291 (PMC10222764; doi:10.3390/nu15102291)
Supplement: Supplementary file 1 [file nutrients-15-02291-s001.zip › nutrients-2375547-supplementary.pdf]

Supplementary Material

**Food survey**
Processing time: approx. 20 min.

[EDP note: welcome text]

Good day!
The Osnabrück University of Applied Sciences is conducting a consumer survey on the topic "Food and Nutrition" as part of a research project. You can support us here with your personal experiences and ideas. The survey will take about 20 min. Thank you for your participation!

Screener

1. How old are you?

[EDP: mandatory field, years as drop-down menu, minimum age 18 years, quotes]

Vintage	Age Groups	%
2001–1995	18–24	9
1994–1985	25–34	15
1984–1975	35–44	15
1974–1965	45–54	17
1964–1949	55–64	18
1950–	>65	26

|__|__|__|__| years.

2. You are …?

[EDP: mandatory field, quotes]

Gender	%
Male	50
Female	50

1 ☐	Male
2 ☐	Female
3 ☐	Divers
−999 ☐	No data

3. In which state do you life?

[EDP: single entry, drop down menu, mandatory field, quote]

Region	Stater	Quote
North	Bremen, Hamburg, Mecklenburg-Vorpommern, Lower Saxony or Schleswig-Holstein	18%
West	North Rhine-Westphalia, Hesse, Rhineland-Palatinate or Saarland	35%
South	Bavaria or Baden-Wurttemberg	29%
East	Berlin, Brandenburg, Saxony, Saxony-Anhalt or Thuringia	18%

1 ☐	Baden-Wurttemberg
2 ☐	Bavaria
3 ☐	Berlin
4 ☐	Brandenburg

5 ☐	Bremen
6 ☐	Hamburg
7 ☐	Hesse
8 ☐	Mecklenburg-Vorpommern
9 ☐	Lower Saxony
10 ☐	North Rhine-Westphalia
11 ☐	Rhineland-Palatinate
12 ☐	Saarland
13 ☐	Saxony
14 ☐	Saxony-Anhalt
15 ☐	Schleswig-Holstein
16 ☐	Thuringia

4. Who is primarily responsible for purchasing groceries in your household?

[EDP: single answer, drop down menu, screenout if F6 = 0]

1 ☐	Mainly myself
2 ☐	Myself and another person
0 ☐	Almost another person→Screenout

5. How often do you consume the following vegetables?

[EDP: single answer, randomized, mandatory field, screenout if all statements F1 = 0]

	**Daily**	**Several Times a Week**	**About Weekly**	**Several Times per Month**	**About Monthly**	**Rarer**	**Never**
Spinach	6 ☐	5 ☐	4 ☐	3 ☐	2 ☐	1 ☐	0 ☐
Arugula	6 ☐	5 ☐	4 ☐	3 ☐	2 ☐	1 ☐	0 ☐
Broccoli	6 ☐	5 ☐	4 ☐	3 ☐	2 ☐	1 ☐	0 ☐
Kohlrabi	6 ☐	5 ☐	4 ☐	3 ☐	2 ☐	1 ☐	0 ☐
Pepper	6 ☐	5 ☐	4 ☐	3 ☐	2 ☐	1 ☐	0 ☐

Main survey: The following questions are about your fresh vegetables buying habits.

6. Which types of cabbage have you frequently eaten in the last year? In case of multiple entries (a maximum of three types of cabbage can be selected), please then rank them from 1 = most frequently eaten to 3 = third most frequently eaten.

[EDP: Randomized, multi choice, max. three entries, ranking top 3]

1 ☐	Cauliflower
2 ☐	White cabbage
3 ☐	Red cabbage/blue cabbage
4 ☐	Chinese cabbage
5 ☐	Kohlrabi
6 ☐	Savoy cabbage
7 ☐	Pak Choi
8 ☐	Broccoli
9 ☐	Kale
10 ☐	Brussels sprouts

7. Where do you buy fresh vegetables most often?

[EDP: single entry, randomized, “other and indeed“ fixed below]

1 ☐	Discount stores (e.g., ALDI, Netto)
2 ☐	Supermarket (e.g., EDEKA, REWE)
3 ☐	Farm store or farmer market
4 ☐	Organic produce shop (e.g., Alnatura, Denns)

5 ☐	I am self-sufficient (e.g., own garden)
6 ☐	Others namely

8. Please select four aspects from the following list that are most important to you when purchasing vegetables? Please then rank them in order from 1 (most important) to 4.

[EDP: multiple entries with max. four mentions, randomize, ranking top 4]

1 ☐	Fresh
2 ☐	Regional origin
3 ☐	Low price
4 ☐	Packaging without plastic
5 ☐	Organic certification
6 ☐	Fair Trade
7 ☐	Flawless appearance
8 ☐	Good taste
9 ☐	Environmentally friendly cultivation

9. How do you rate the following statements? Please rate your opinion on a scale of “Does not apply at all“ to “Applies completely“

[EDP: randomized–block by block

	**Does Not Apply at All**	**Rather Not Applicable**	**Part/Part**	**More Likely to Apply**	**Fully Applies**
Pleasure plays the most important role in my food choice.	−2 ☐	−1 ☐	0 ☐	+1 ☐	+2 ☐
For me, a good taste is more important than the health value of a food.	−2 ☐	−1 ☐	0 ☐	+1 ☐	+2 ☐
I am willing to compromise on the taste of a food if the product is healthy.	−2 ☐	−1 ☐	0 ☐	+1 ☐	+2 ☐
Good food and drink play a big role in my life.	−2 ☐	−1 ☐	0 ☐	+1 ☐	+2 ☐
[EDP: Information Needs/Security]					
When shopping, I look for certifications and labels (e.g., organic, preservative-free, gluten-free) because they give me a sense of security.	−2 ☐	−1 ☐	0 ☐	+1 ☐	+2 ☐
I often read the information on the packaging of products.	−2 ☐	−1 ☐	0 ☐	+1 ☐	+2 ☐
It is important to me to have trustworthy sources of information.	−2 ☐	−1 ☐	0 ☐	+1 ☐	+2 ☐
I like to try new and innovative foods.	−2 ☐	−1 ☐	0 ☐	+1 ☐	+2 ☐
[EDP: Convenience]					
My professional life is often very demanding, so I´m glad when I can resort to semi-finished or finished products.	−2 ☐	−1 ☐	0 ☐	+1 ☐	+2 ☐
I find ready meals are helpful addition to the kitchen.	−2 ☐	−1 ☐	0 ☐	+1 ☐	+2 ☐
I prefer to cook dishes that are easy to prepare.	−2 ☐	−1 ☐	0 ☐	+1 ☐	+2 ☐

10. Have you heard of “biofortified foods“ in context of food or nutrition?

[EDP: single entry]

1 ☐	Yes
2 ☐	No
0 ☐	I am not sure

11. Please select characteristics from the following list that you associate with biofortified vegetables (even if you have not heard of them before).

[EDV: multiple choice, randomize, options *vegetables enriched with nutrients during cultivation* and *vegetables enriched with nutrients during processing* fix in order]

"In my opinion, biofortified vegetables are…"

1 ☐	… vegetables from organic farming.
2 ☐	… vegetables enriched with nutrients during cultivation.
3 ☐	… vegetables enriched with nutrients during processing.
4 ☐	… produced without the use of chemical pesticides.
5 ☐	… particularly rich in essential minerals and vitamins.
6 ☐	… vegetables fertilized only with organic fertilizers.
7 ☐	… vegetables with increased health value.

12. We would like to know more about your attitudes towards the topic of "health and nutrition". How would you rate the following statements? Please rate your opinion on a scale from "not at all true" to "strongly true".

[EDP: randomized–block by block]

	Does Not Apply at All	Rather Not Applicable	Part/Part	More Likely to Apply	Fully Applies
[EDP: Health promotion]					
I consciously pay attention to a varied and balanced diet with lots of vegetables.	−2 ☐	−1 ☐	0 ☐	+1 ☐	+2 ☐
I consciously make sure that I walk for at least 30 min a day.	−2 ☐	−1 ☐	0 ☐	+1 ☐	+2 ☐
My physical fitness is very important to me.	−2 ☐	−1 ☐	0 ☐	+1 ☐	+2 ☐
I think a lot about my diet because it´s important for my health.	−2 ☐	−1 ☐	0 ☐	+1 ☐	+2 ☐
[EDP: Health care]					
I regular take cancer screening and other health examinations (e.g., skin cancer/colon cancer/breast cancer/glaucoma).	−2 ☐	−1 ☐	0 ☐	+1 ☐	+2 ☐
I have my blood work done regularly.	−2 ☐	−1 ☐	0 ☐	+1 ☐	+2 ☐
I regularly take nutritional supplements to maintain my health.	−2 ☐	−1 ☐	0 ☐	+1 ☐	+2 ☐
I try to prevent diseases (e.g., by vaccinations/annual check-ups at the dentist).	−2 ☐	−1 ☐	0 ☐	+1 ☐	+2 ☐

The following questions revolve around the topic of nutrients in food.

13. In this selection we would like to talk to you about nutrients (essential minerals, vitamin and amino acids). Which of the following nutrients have you heard of in connection with nutrition?

[EDP: randomize, multiple choice, fix "none of the above" below]

1 ☐	Fluorine
2 ☐	Vitamin C
3 ☐	Beta-carotene
4 ☐	Cysteine
5 ☐	Methionine
6 ☐	Zinc
7 ☐	Iron
8 ☐	Iodine
9 ☐	Molybdenum
10 ☐	Selenium
−999 ☐	None of the above

14. Have you ever heard of "functional food" in the context of food or nutrition?

[EDP: single entry]

1 ☐	Yes
2 ☐	No
0 ☐	I am not sure

15. Please select from the following list properties that you associate with "functional foods" (even if you have not heard of them before).

[EDP: multiple answers, randomize, Fix option *vegetables enriched with nutrients during cultivation* and *vegetables enriched with nutrients during processing* order one after the other]

"In my opinion, functional foods are …"

1 ☐	… foods that can help prevent diseases.
2 ☐	… foods that are enriched with nutrients during cultivation.
3 ☐	… foods that are enriched with nutrients during processing.
4 ☐	… foods that are particularly rich in essential minerals and vitamins.
5 ☐	… foods with increased health value.
6 ☐	… foods that are easy and quick to prepare.
7 ☐	… foods that help improve physical performance.
8 ☐	… foods that can be kept for a long time without spoiling.
9 ☐	… foods that have a positive effect on well-being.

16. When you think about the effect of iron on your body, how do you rate it?

**Clearly Negative Effect**	**Rather Negative Effect**	**Undecided**	**Rather Positive Effect**	**Clearly Positive Effect**
−2 ☐	−1 ☐	0 ☐	+1 ☐	+2 ☐

17. Off the top of my head, what health effects dies a deficiency of iron bring to the human body?

[Editing Note: Feel free to respond in keyword form.]

18. Which of the following health impairments do you think are due to an iron deficiency?

[EDP: multiple answers, randomize, fix "I don´t know" below]

1 ☐	Anemia or blood deficiency
2 ☐	Taste disorder
3 ☐	Cold hands and feeds
4 ☐	Immune system disorder
5 ☐	Learning and memory difficulties
6 ☐	Fatigue
7 ☐	Shortness of breath
8 ☐	Dizziness
9 ☐	Tingling in the legs
10 ☐	Brittle fingernales
11 ☐	Fast and irregular heartbeat
0 ☐	I do not know

19. Do you know people in your close family environment (e.g., parents, siblings, spouse or even yourself) who are affected by the consequences of a temporary iron deficiency?

[EDP: single answers]

3 ☐	Yes, several people
2 ☐	Yes, one person
1 ☐	I have never dealt with the subject
0 ☐	I do not know

20. In your opinion, what contributions do the following foods make to your personal iron supply?

[EDP: randomize]

	**No Contribution**	**Minor Contribution**	**Average Contribution**	**Very Large Contribution**
Fish and seafood	1 ☐	2 ☐	3 ☐	4 ☐

Fruit	1 ☐	2 ☐	3 ☐	4 ☐
Vegetables	1 ☐	2 ☐	3 ☐	4 ☐
Cereal, and cereal products	1 ☐	2 ☐	3 ☐	4 ☐
Milk, and dairy products	1 ☐	2 ☐	3 ☐	4 ☐
Meat, and meat products	1 ☐	2 ☐	3 ☐	4 ☐
Drinking water, and non-alcoholic beverages	1 ☐	2 ☐	3 ☐	4 ☐
Confectionery and snacks	1 ☐	2 ☐	3 ☐	4 ☐
Legumes (e.g., beans, peas)	1 ☐	2 ☐	3 ☐	4 ☐
Seeds, and nuts	1 ☐	2 ☐	3 ☐	4 ☐

21. Which of the following products do you use or would you use to prevent or counteract a possible iron deficiency?

[EDP: multiple answers, randomize, fix *other foods* option below and *I don´t pay attention to it* above]

1 ☐	I do not pay special attention
2 ☐	Meat (e.g., beef)
3 ☐	Sausages (e.g liver sausage)
4 ☐	Cereal products (e.g. oat flakes, wheat bran, millet flakes)
5 ☐	Legumes (e.g., beans, peas, lentils)
6 ☐	Seeds and nuts (e.g., pumpkin seeds, pine nuts)
7 ☐	Vegetables
8 ☐	Dietary supplements
9 ☐	Foods rich in vitamin C to improve iron absorption
10 ☐	Foods that are enriched with iron (e.g. fruit juices, cornflakes)
0 ☐	Other foods that are rich in iron and namely: ___________________________

22. The production and processing of foods can increase their iron content. For which foods do you find an increase appealing in order to absorb additional iron through your diet?

[EDP: multiple answers, randomize, fix options *Fruit* and *processed fruit* AND *vegetables* and *processed vegetables* order one after the other, fix, “With no food” at the bottom]

1 ☐	Meat, and meat product
2 ☐	Milk, and dairy products
3 ☐	Cereals, and cereal products
4 ☐	Fresh vegetables
5 ☐	Processed vegetables
6 ☐	Fresh fruits
7 ☐	Processed fruits
8 ☐	Confectionery, and snacks
9 ☐	Non alcoholic beverages (mineral water, juices, etc.)
10 ☐	Alcoholic beverages
11 ☐	Egg, and egg products
12 ☐	Ready to eat products
0 ☐	With no food

[EDP: Split Ballot→Goup A: n = 500 continued with F 23, Groupe B: n = 500→continued with F25, random allocation 50:50]

GROUP A

Concept test: introduction of new iron products

In the following section, we would like to introduce you to new vegetable products that contain more than twice the usual amount of the iron due to special cultivation. Iron is a vital trace element that plays decisive role in maintaining human health.

23. Regular consumption of iron-rich vegetables can have certain effects. How strongly would you personally orient yourself to the following additional information when purchasing vegetables products? Please rate your assessment on a scale from “not at all” to “very strongly”.

[EDP: filter: group A, randomize]

	Not at All	Rather Not	Partly/ Partly	Rather Strong	Very Strong
Contributes to normal cognitive function, such as memory and learning.	−2 ☐	−1 ☐	0 ☐	+1 ☐	+2 ☐
Contributes to normal energy metabolism	−2 ☐	−1 ☐	0 ☐	+1 ☐	+2 ☐
Contributes to normal formation of red blood cells and hemoglobin.	−2 ☐	−1 ☐	0 ☐	+1 ☐	+2 ☐
Contributes to normal oxygen transport in the body.	−2 ☐	−1 ☐	0 ☐	+1 ☐	+2 ☐
Contributes to normal functioning of the immune system.	−2 ☐	−1 ☐	0 ☐	+1 ☐	+2 ☐
Helps reduce fatigue and tiredness.	−2 ☐	−1 ☐	0 ☐	+1 ☐	+2 ☐
Has a function in cell division.	−2 ☐	−1 ☐	0 ☐	+1 ☐	+2 ☐

[EDP: show image and text on a separate page]

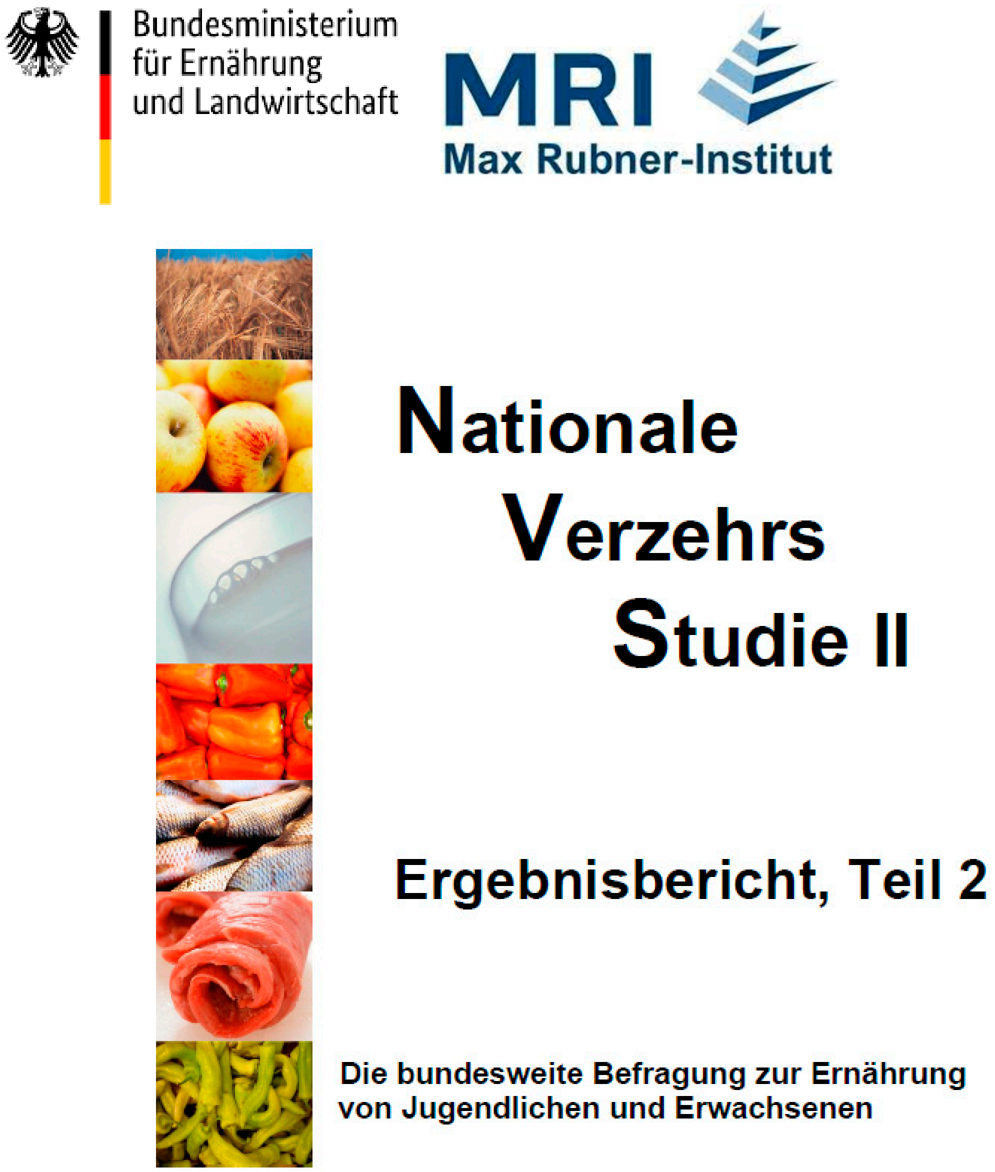

The National Consumption Study II shows that the iron supply in Germany is not optimal. Overall, more than one third of adolescents and adults do not reach the recommended daily intake for iron.
Women under the age of 50 are particularly likely to be undersupplied with iron. On average, young women up to the age of 24 consume only half the recommended amount of iron. Women´s iron requirements are also higher during pregnancy and lactation.
Common symptoms of iron deficiency are tiredness, fatigue, lack of concentration and malaise. A deficiency over a longer period of time can lead to iron deficiency anemia.

It is therefore important to ensure an adequate supply of iron. Iron-rich vegetables can contribute to this.

24. Vegetables can contain more than twice as much iron as usual due to a special cultivation. Various types of vegetables come into consideration for this increase in iron content. How much do the following iron-rich vegetables appeal to you? Please rate your opinion on a scale from "Doesn´t appeal to me at all" to "Appeals to me a lot".
[EDP: filter: group A only, randomize]

	**Does not Appeal to Me at All**	**Rather Does not Appeal to Me**	**Part/Part**	**Appeals to Me More**	**Appeals to Me very Much**
Spinach 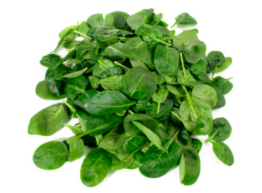	−2 ☐	−1 ☐	0 ☐	+1 ☐	+2 ☐
Arugula 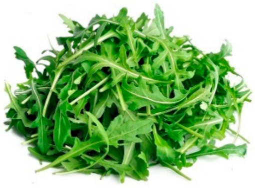	−2 ☐	−1 ☐	0 ☐	+1 ☐	+2 ☐
Broccoli 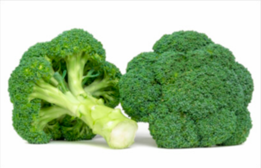	−2 ☐	−1 ☐	0 ☐	+1 ☐	+2 ☐
Bell pepper 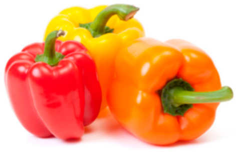	−2 ☐	−1 ☐	0 ☐	+1 ☐	+2 ☐
Kohlrabi 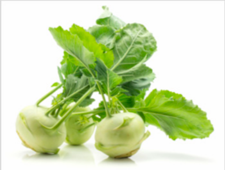	−2 ☐	−1 ☐	0 ☐	+1 ☐	+2 ☐

GROUP B

25. Vegetables can contain more than twice as much iron as usual due to a special cultivation. Various types of vegetables come into consideration for this increase in iron content. How much do the following iron-rich vegetables appeal to you? Please rate your opinion on a scale from "Doesn´t appeal to me at all" to "Appeals to me a lot".
[EDP: filter: group A only, randomize]

	Does not Appeal to Me at All	Rather Does not Appeal to Me	Part/Part	Appeals to Me More	Appeals to Me Very Much
Spinach 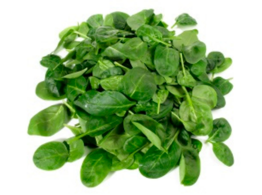	−2 ☐	−1 ☐	0 ☐	+1 ☐	+2 ☐
Arugula 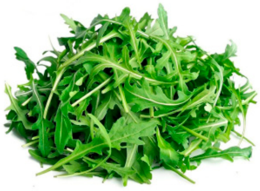	−2 ☐	−1 ☐	0 ☐	+1 ☐	+2 ☐
Broccoli 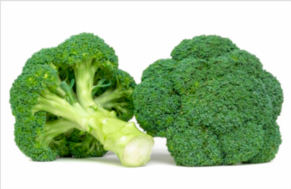	−2 ☐	−1 ☐	0 ☐	+1 ☐	+2 ☐
Bell pepper 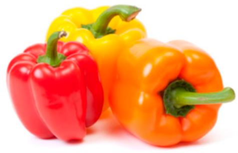	−2 ☐	−1 ☐	0 ☐	+1 ☐	+2 ☐
Kohlrabi 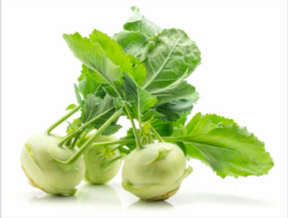	−2 ☐	−1 ☐	0 ☐	+1 ☐	+2 ☐

26. Regular consumption of iron-rich vegetables can have certain effects. How strongly would you personally orient yourself to the following additional information when purchasing vegetables products? Please rate your assessment on a scale from "not at all" to "very strongly".
[EDP: filter: group A, randomize]

	Not at All	Rather Not	Partly/ Partly	Rather Strong	Very Strong
Contributes to normal cognitive function, such as memory and learning.	−2 ☐	−1 ☐	0 ☐	+1 ☐	+2 ☐
Contributes to normal energy metabolism	−2 ☐	−1 ☐	0 ☐	+1 ☐	+2 ☐
Contributes to normal formation of red blood cells and hemoglobin.	−2 ☐	−1 ☐	0 ☐	+1 ☐	+2 ☐
Contributes to normal oxygen transport in the body.	−2 ☐	−1 ☐	0 ☐	+1 ☐	+2 ☐
Contributes to normal functioning of the immune system.	−2 ☐	−1 ☐	0 ☐	+1 ☐	+2 ☐
Helps reduce fatigue and tiredness.	−2 ☐	−1 ☐	0 ☐	+1 ☐	+2 ☐
Has a function in cell division.	−2 ☐	−1 ☐	0 ☐	+1 ☐	+2 ☐

ALL

27. Which of these five vegetables appeals to you most, if they are particularly rich in iron? If you have multiple answers (up to a maximum of 3 vegetables), please rank them in order from 1 (appeals to me the most) to a maximum of 3.

[EDP: compulsory question, randomize, multiple answers max. 3 entries; ranking top 3]

1 ☐	Spinach
2 ☐	Arugula
3 ☐	Kohlrabi
4 ☐	Broccoli
5 ☐	Bell pepper

28. In what form would iron-rich (name of vegetable) [EDP: top 1 selection from F27] particularly appeal to you:

[EDP: mandatory question, single answer filter F27 = 1, 3 or 4]

3 ☐	As fresh, unprocessed …. (name of vegetables) [EDP: note insert top 1 selection from F27]
2 ☐	As processed, frozen (name of vegetable [EDP Top 1 note insert top 1 selection from F27]
1 ☐	In the form of finished products (ready-to-eat dishes)

29. In what form would iron-rich arugula appeal to you most?

[EDP: mandatory question, single-entry filter F27 = 2]

3 ☐	As fresh, unprocessed arugula
2 ☐	As arugula pesto
1 ☐	As an ingredient in finished products (edible dishes)

30. Which iron-rich pepper variety would appeal to you most?

[EDP: mandatory question, single answer, randomize fix, "I don´t care about the type of pepper" at the bottom, Filter F27 = 5]

1 ☐	Red pepper
2 ☐	Orange pepper
3 ☐	Yellow pepper
4 ☐	Green pepper
0 ☐	I don´t care about the pepper variety.

31. Why do you find (selected color) [EDP: insert selection from F27] particularly appealing for increasing the iron content?

[EDP: mandatory question, multiple choice, randomize, F27 = 5]

1 ☐	Because I like this variety of bell peppers the best.
2 ☐	Because this variety of bell peppers looks the most beautiful.
3 ☐	Because this variety of bell peppers goes well with increased iron content.
4 ☐	Because this variety of bell peppers is particularly healthy.

[EDP: 5way Split of the 1000 people into groups of 200 (A, B, C, D and E)–blind acceptance test]

32. (A) When shopping, if you had a choice of a common (name of vegetable) [EDP: insert top 1 choice from F27] and a particularly iron-rich (name of vegetable) [EDP: insert top 1 choice from F27], which product would you choose?

[EDP: group A only, single answer, show name of vegetable in scale label]

**Most Certainly Common (Name of Vegetable)**					**I Am Undecided**					**Most Certainly Especially Iron-Rich (Name of Vegetable)**
☐0	☐ 1	☐ 2	☐ 3	☐ 4	☐ 5	☐ 6	☐ 7	☐ 8	☐ 9	☐ 10

(B) Iron-rich (name of vegetable) [EDP: insert top 1 selection from F27] is also characterized by increased vitamin C content. Vitamin C promotes iron absorption in the body and thus supports an improvement in iron supply.

When shopping, if you had a choice of a common (name of vegetable) [EDP: insert top 1 choice from F27] and a particularly iron-rich (name of vegetable) [EDP: insert top 1 choice from F27], which product would you choose?

[EDP: group B only, single answer, show name of vegetable in scale label]

**Most Certainly Common (Name of Vegetable)**					**I Am Undecided**					**Most Certainly Especially Iron-Rich (Name of Vegetable)**
☐0	☐ 1	☐ 2	☐ 3	☐ 4	☐ 5	☐ 6	☐ 7	☐ 8	☐ 9	☐ 10

(C) Iron-rich (name of vegetable) [EDP: insert top 1 selection from F27] is also characterized by increased content of iodine. Iodine is another vital trace element that contributes, among other things, to the normal functioning of the thyroid gland.

When shopping, if you had a choice of a common (name of vegetable) [EDP: insert top 1 choice from F27] and a particularly iron-rich (name of vegetable) [EDP: insert top 1 choice from F27], which product would you choose?

[EDP: group C only, single answer, show name of vegetable in scale label]

**Most Certainly Common (Name of Vegetable)**					**I Am Undecided**					**Most Certainly Especially Iron-Rich (Name of Vegetable)**
☐0	☐ 1	☐ 2	☐ 3	☐ 4	☐ 5	☐ 6	☐ 7	☐ 8	☐ 9	☐ 10

(D) Iron-rich (name of vegetable) [EDP: insert top 1 selection from F27] is less contaminated with undesirable substances such as cadmium and nitrate due to its special cultivation.

When shopping, if you had a choice of a common (name of vegetable) [EDP: insert top 1 choice from F27] and a particularly iron-rich (name of vegetable) [EDP: insert top 1 choice from F27], which product would you choose?

[EDP: group D only, single answer, show name of vegetable in scale label]

**Most Certainly Common (Name of Vegetable)**					**I Am Undecided**					**Most Certainly Especially Iron-Rich (Name of Vegetable)**
☐0	☐ 1	☐ 2	☐ 3	☐ 4	☐ 5	☐ 6	☐ 7	☐ 8	☐ 9	☐ 10

(E) When growing iron-rich (name of vegetable) [EDP: insert top 1 selection from F27], less nitrogen is fertilized and fewer crop losses occur. This results in less environmental impact and food losses is the production of the vegetables.

When shopping, if you had a choice of a common (name of vegetable) [EDP: insert top 1 choice from F27] and a particularly iron-rich (name of vegetable) [EDP: insert top 1 choice from F27], which product would you choose?

[EDP: group E only, single answer, show name of vegetable in scale label]

Most Certainly Common (Name of Vegetable)					I Am Undecided					Most Certainly Especially Iron-Rich (Name of Vegetable)
☐0	☐ 1	☐ 2	☐ 3	☐ 4	☐ 5	☐ 6	☐ 7	☐ 8	☐ 9	☐ 10

[EDP: blind price test for top 3 vegetables from question 27for each vegetable type, the price is queried in 5-way split. Participants are shown a price per preferred vegetable according to the top 3 vegetable choices].

33. Imagine the following situation: While shopping, you see (name of vegetable) [EPD: insert selection from F27], which is particularly rich in iron. How likely is that you would buy this (name of vegetable) [EDP insert selection from F27] if the price for (packaging unit of the vegetable) is € X.XX.

For comparison: A (gram mage vegetable) with normal (name of vegetable) [EDP: insert selection from F27] cost on average (price of vegetable column 0).

Group	0 (Reference)	1 + € 0.10	2 + € 0.20	3 + € 0.30	4 + € 0.40	5 + € 0.50
Price spinach 250 g 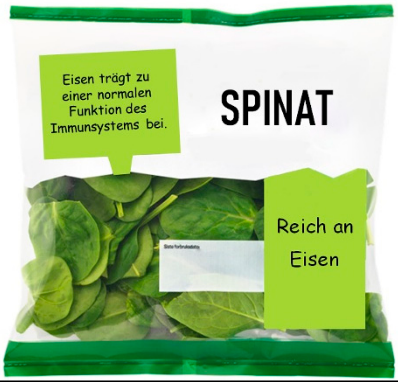	1.79	1.89	1.99	2.09	2.19	2.29
Price arugula 125 g 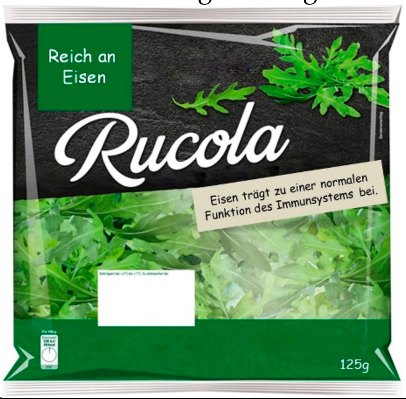	0.89	0.99	1.09	1.19	1.29	1.39
Price broccoli pcs.	1.19	1.29	1.39	1.49	1.59	1.69

<table>
<tr><td>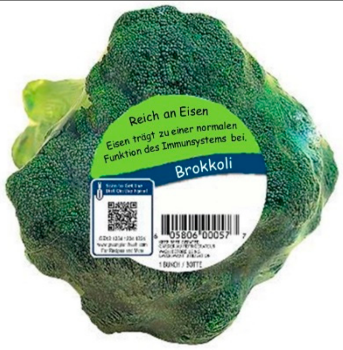
</td><td></td><td></td><td></td><td></td><td></td><td></td></tr>
<tr><td>Price pepper 3 pcs.
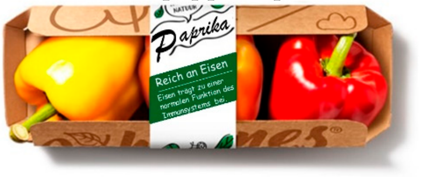
</td><td>1.09</td><td>1.19</td><td>1.29</td><td>1.39</td><td>1.49</td><td>1.59</td></tr>
<tr><td>Price kohlrabi pcs.
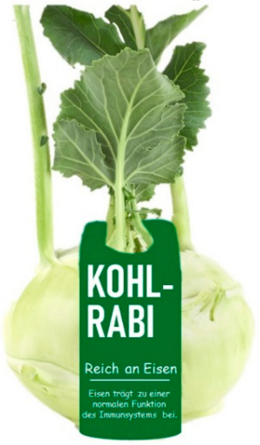
</td><td>0.49</td><td>0.59</td><td>0.69</td><td>0.79</td><td>0.89</td><td>0.99</td></tr>
<tr><td>Selection</td><td></td><td>10 scale</td><td>10 scale</td><td>10 scale</td><td>10 scale</td><td>10 scale</td></tr>
</table>

That I buy particularly iron-rich (name of vegetable) [EPD: insert selection from F27, in case of multiple entries in F27 the question block must appear several times] at a price of X.XX€ is…

**Not at All Likely**									**Highly Probable**
1	2	3	4	5	6	7	8	9	10

34. Please select number 2 from the selection below if you would like to continue the survey.

    [EDP: Attention question: abort if value ≠ 2, quality fail]

**0**	**1**	**2**	**3**	**4**	**5**	**6**	**7**	**8**	**9**	**10**
0	1	2	3	4	5	6	7	8	9	10

35. Imagine you are in the supermarket and want to buy (name of vegetable) [EDP: insert selection from F27]. How would you rate the following labeling on your purchase on a scale from “Doesn´t appeal to me at all” to “Appeals to me a lot”?

    [EDP: Randomize, fix answer choice “Rich in iron” and “Rich in iron and vitamin C among each other]

	Does Not Appeal to Me at All	Rather Does Not Appeal to Me	Part/Part	Appeals to Me More	Appeals to Me Very Much
High iron content	−2 ☐	−1 ☐	0 ☐	+1 ☐	+2 ☐
Iron source	−2 ☐	−1 ☐	0 ☐	+1 ☐	+2 ☐
Enriched with iron	−2 ☐	−1 ☐	0 ☐	+1 ☐	+2 ☐
Biofortified with iron	−2 ☐	−1 ☐	0 ☐	+1 ☐	+2 ☐
Rich in iron	−2 ☐	−1 ☐	0 ☐	+1 ☐	+2 ☐
Rich in iron and vitamin C	−2 ☐	−1 ☐	0 ☐	+1 ☐	+2 ☐
With high content of bioavailable iron	−2 ☐	−1 ☐	0 ☐	+1 ☐	+2 ☐

These questions are about your food consumption patterns.

36. We would like to know more about your attitudes toward buying and consuming food. How would you rate the following statements? Please rate your opinion on a scale from “Does not apply at all” to “Fully applies”.

[EDP: randomize]

	Does Not Apply at All	Rather Not Applicable	Part/Part	More Likely to Apply	Fully Applies
	[sustainability]				
In many of my decisions, I consider the potential environmental impact of my actions.	−2 ☐	−1 ☐	0 ☐	+1 ☐	+2 ☐
I actively try to avoid packaging waste, especially plastic packaging, through my shopping.	−2 ☐	−1 ☐	0 ☐	+1 ☐	+2 ☐
When shopping, I pay attention to the compability of the products with regard to the environment, animal welfare and fair wages.	−2 ☐	−1 ☐	0 ☐	+1 ☐	+2 ☐
I look specifically for the origin and prefer regional products.	−2 ☐	−1 ☐	0 ☐	+1 ☐	+2 ☐
I often buy organic food	−2 ☐	−1 ☐	0 ☐	+1 ☐	+2 ☐
	[naturalness]				
I often eat fresh fruits and vegetables.	−2 ☐	−1 ☐	0 ☐	+1 ☐	+2 ☐
I often buy food that has been processed in a protective way and as little as possible-	−2 ☐	−1 ☐	0 ☐	+1 ☐	+2 ☐
I cook for myself (and my family) several times a week.	−2 ☐	−1 ☐	0 ☐	+1 ☐	+2 ☐
I prefer food without additives and colorants.	−2 ☐	−1 ☐	0 ☐	+1 ☐	+2 ☐

This section is about health aspects of food consumption.

37. Please select from the following list the four most important characteristics that characterize healthy (name of vegetable) [EDP: insert selection from F27] for you? Please then rank them in order from 1 (most important) to 4.

[EDP: multiple responses with four mentions, ranking top 4]

1 ☐	Produced without the use of pesticides
2 ☐	Grown away from industry and traffic
3 ☐	Rich in vitamins
4 ☐	Low allergen
5 ☐	Rich in minerals and trace elements
6 ☐	Free from pollutants such as heavy metals
7 ☐	Free from pesticide residues
8 ☐	Produced without the use of fertilizers

38. How often do you take nutrients such as iron, magnesium, and vitamin C as supplements?
[EDP: single entry]

7 ☐	Daily
6 ☐	Several times a week
5 ☐	Once a week
4 ☐	Several times per month
3 ☐	Monthly
2 ☐	Once in 3 months
1 ☐	Rare
0 ☐	Never

39. Which of the following would you be more likely to attribute to an iron-rich vegetable such as (name of vegetable) [EDP: insert top 1 from F27], a functional food with added iron, or iron-containing dietary supplements?
[EDP: single entry, randomize]

	**Rather Iron Rich Vegetables [EDP: Show Product & Image from F27 Top 1]**	**Rather Functional Food with Added Iron**	**Rather Iron Dietary Supplements**
		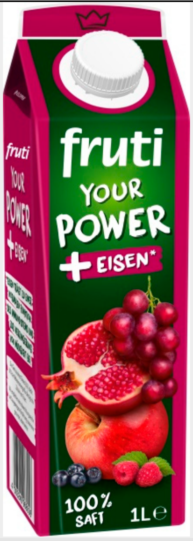	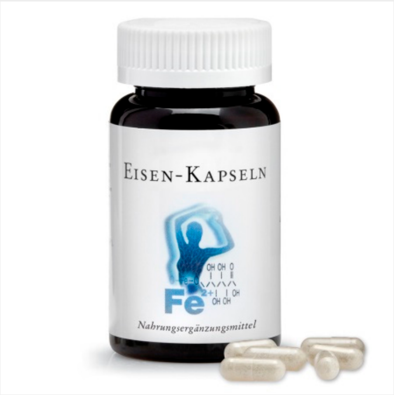
Healthy	1☐	2☐	3☐
Trustworthy	1☐	2☐	3☐
Safe	1☐	2☐	3☐
Effective	1☐	2☐	3☐
Natural	1☐	2☐	3☐
Environmentally friendly	1☐	2☐	3☐
Costly	1☐	2☐	3☐
Dosability	1☐	2☐	3☐
Everyday suitability	1☐	2☐	3☐
For a good iron supply I prefer	1☐	2☐	3☐

40. How often do you consume enriched foods such as fruity juices with added vitamin C, probiotic yogurt, bread with omega-3 fatty acids, or caffeinated energy drinks?
[EDP: single entry]

7 ☐	Daily
6 ☐	Several times a week
5 ☐	Once a week

4 ☐ Several times per month
3 ☐ Monthly
2 ☐ Once in 3 months
1 ☐ Rarer
0 ☐ Never

41. Do you have an iron deficiency?

1 ☐ Yes
2 ☐ Probably, but I am not sure
3 ☐ No
0 ☐ I do not know

42. Are you taking any medications to address your iron deficiency?
[EDP: only if F37 = 1 or 2]

1 ☐ Yes
0 ☐ No

43. Are you aiming for a special diet?
[EDP: single answer]

1 ☐ I make a conscious effort to eat less meat
2 ☐ Vegan diet
3 ☐ Vegetarian diet
4 ☐ Allergy adapted diet
5 ☐ No special diet
6 ☐ I follow a different diet and that is:____

Sociodemographic criteria

You have almost made it. Finally, we would like to ask you a few questions about yourself.

44. How would you describe where you live?
[EDV: mandatory question, single answer]

1 ☐ In the city center
2 ☐ At the edge of the city
3 ☐ Rural

45. What is your highest school-leaving qualification?
[EDP: mandatory question, single answer]

1 ☐ Still pupil
2 ☐ Elementary/secondary school
3 ☐ Middle school
4 ☐ Baccalaureate
5 ☐ Master craftsman/technician/technical college degree
6 ☐ University
0 ☐ Without degree
−999 ☐ Other and namely

46. Which of the following best describes your current job situation?
[EDP single answer]

1 ☐ Pupil
2 ☐ Trainee
3 ☐ Student
4 ☐ Fully employee
5 ☐ Part-time employee
6 ☐ Pension
7 ☐ Housewife/househusband

8 ☐ Unemployed	
−999 ☐ No specification	

47. How many people in total are in your household?
[EDP: single entry, show pull-down list with number of people]

1 ☐ 1 person	
2 ☐ 2 people	
3 ☐ 3 people	
4 ☐ 4 people	
5 ☐ 5 people	
6 ☐ More than 5 people	
−999 ☐ No specification	

48. Number of children under 12 years
[EDP only if F47 > 1]
|__|__| children

49. In which of the following classes would you place your net monthly household income?
[EDP: single answer]

1 ☐	Under €1300
2 ☐	€1300–€2600
3 ☐	€2600–€3600
4 ☐	€3600–€5000
5 ☐	Over €5000
−999 ☐	Not specified
